# A network analysis of global cephalopod trade

**DOI:** 10.1038/s41598-021-03777-9

**Published:** 2022-01-10

**Authors:** Andres Ospina-Alvarez, Silvia de Juan, Pablo Pita, Gillian Barbara Ainsworth, Fábio L. Matos, Cristina Pita, Sebastián Villasante

**Affiliations:** 1 Mediterranean Institute for Advanced Studies, IMEDEA (UIB-CSIC), C/Miquel Marques 21, CP 07190 Esporles, Balearic Islands Spain; 2 Institute of Marine Sciences ICM (CSIC), Passeig Maritim de la Barceloneta 37, CP 08003 Barcelona, Spain; 3 CRETUS Department of Applied Economics, University of Santiago de Compostela, Campus Sur, Santiago de Compostela, A Coruña Spain; 4 Faculty of Business Administration and Management, University of Santiago de Compostela, Santiago de Compostela, A Coruña Spain; 5 Department of Environment and Planning, CESAM-Centre for Environmental and Marine Studies, University of Aveiro, Aveiro, Portugal; 6 International Institute for Environment and Development (IIED), London, UK

**Keywords:** Environmental economics, Environmental economics

## Abstract

The global trade in cephalopods is a multi-billion dollar business involving the fishing and production of more than ten commercially valuable species. It also contributes, in whole or in part, to the subsistence and economic livelihoods of thousands of coastal communities around the world. The importance of cephalopods as a major cultural, social, economic, and ecological resource has been widely recognised, but research efforts to describe the extent and scope of the global cephalopod trade are limited. So far, there are no specific regulatory and monitoring systems in place to analyse the traceability of the global trade in cephalopods at the international level. To understand who are the main global players in cephalopod seafood markets, this paper provides, for the first time, a global overview of the legal trade in cephalopods. Twenty years of records compiled in the UN COMTRADE database were analysed. The database contained 115,108 records for squid and cuttlefish and 71,659 records for octopus, including commodity flows between traders (territories or countries) weighted by monetary value (USD) and volume (kg). A theoretical network analysis was used to identify the emergent properties of this large trade network by analysing centrality measures that revealed key insights into the role of traders. The results illustrate that three countries (China, Spain, and Japan) led the majority of global market movements between 2000 and 2019. Based on volume and value, as well as the number of transactions, 11 groups of traders were identified. The leading cluster consisted of only eight traders, who dominated the cephalopod market in Asia (China, India, South Korea, Thailand, and Vietnam), Europe (the Netherlands, and Spain), and the USA. This paper identifies the countries and territories that acted as major importers or exporters, the best-connected traders, the hubs or accumulators, the modulators, the main flow routes, and the weak points of the global cephalopod trade network over the last 20 years. This knowledge of the network is crucial to move towards an environmentally sustainable, transparent, and food-secure global cephalopod trade.

## Introduction

**Figure 1:**
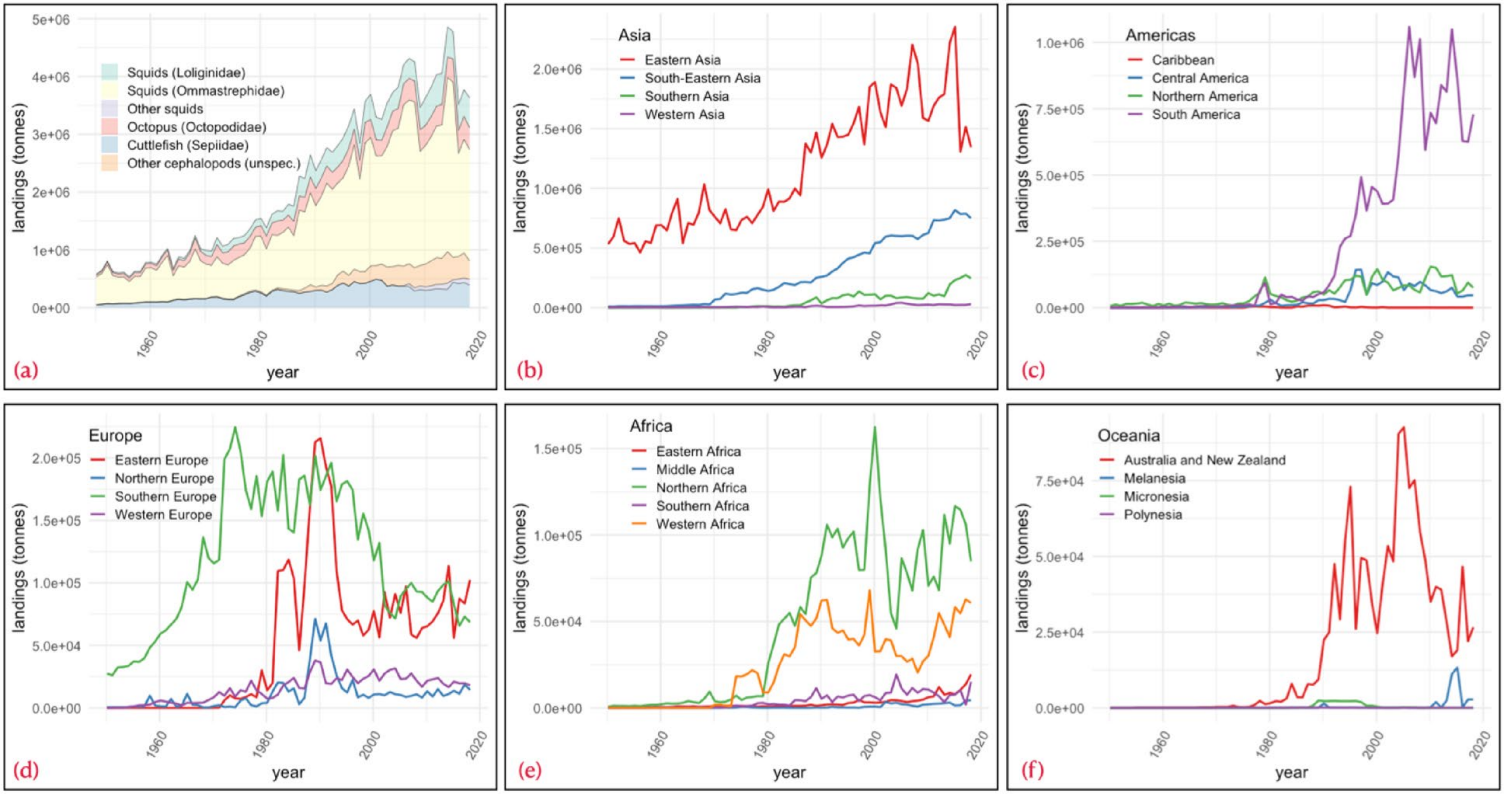
Time series of cephalopod landings by (**a**) taxonomic group and (**b** to **f**) by continents and sub-continents. Values are given in tonnes. (Data source: FAO^2^. The figure was created with R^12^ (https://cran.r-project.org) package “ggplot2” v.3.2.1^13^ (https://ggplot2.tidyverse.org).

Cephalopods account for around 2.5% of seafood production. Landings have increased in relative terms by 416% since 1961 to reach an all-time maximum of around 4 million tonnes in 2013, before dropping to around 3 million tonnes in 2019 (Fig. 1a). East Asia and South America, led by China and Peru, drive the increase in production, while Japan has halved its cephalopod production over the past 50 years^1,2^ (Fig. 1b,c). Despite an evident global trend of declining cephalopod catches since 2013, their commercialisation in volume (tonnes) and monetary value (USA dollars, hereafter USD) has followed a steady increasing trend, by sixfold in volume and 14-fold in USD, since the 1950s. East Asia and South America (particularly Peru and Argentina, including Malvinas/Falkland Islands) have concentrated the highest production of volume in the last 20 years, staying in the same predominant position since the 80s (Fig. 1b,c). Regarding cephalopod market value, Asian (China, Japan, Thailand, Republic of Korea (South Korea), Vietnam, India), European (Spain, Italy), African (Morocco) and North American (the USA) countries hold the 10 most important fishing fleets in the world^2^.

Considering relevant stocks of squid and octopus, worldwide catches of shortfin squid (*Illex* spp., see family Ommastrephidae in Fig. 1a) fell from 850,000 tonnes in 2014 to 200,000 tonnes or less in recent years. Shortfin squid catches in the Malvinas/Falkland Islands have been low (43,400 t) in recent years, and the year 2019 marked the third consecutive year of slow recovery of the South Patagonian stock after its extremely low abundance observed in 2016^3^. Non-favourable oceanographic conditions for stock migrations and intense activity of foreign fishing fleets (China, Japan, Republic of Korea, Taiwan, Spain) are key factors affecting the current abundance of the stock^3,4^ (Fig. 1a,b,d). The United Kingdom’s departure from the European Union (Brexit) in 2020 has also increased uncertainty within the cephalopod market because the structure and dynamic of the global squid trade are expected to be seriously altered. For example, squid producers and traders from the Malvinas/Falkland Islands may lose access to the highly relevant Spanish market. This would affect the profitability of the Spanish fishing fleet based in the Port of Vigo, for which the *Illex* fishery generates €200 million (c.a. USD$ 235 million) per year^5^. Conversely, Peruvian squid landings are estimated to have reached record levels in 2019. The Chinese market is currently open to Peru; therefore, larger volumes may be exported. Landings of Japanese flying squid (*Todarodes pacificus,* see family Ommastrephidae in Fig. 1a) were high in 2019, after some years of declining catches; while squid stocks in the USA waters are not overfished^6^ (Fig. 1c). Meanwhile, octopus landings have recently decreased in the most important supplier countries (Morocco and Mauritania in Northern Africa; Fig. 1e), which influences global supply for this group. Morocco and Mauritania are currently implementing more restrictive management measures to protect their octopus fisheries resource^7^. The lowest fishery landings come from Oceania, where the main players are Australia and New Zealand. These two countries have experienced large fluctuations with a marked downward trend since mid-2005 (Fig. 1f).

**Figure 2:**
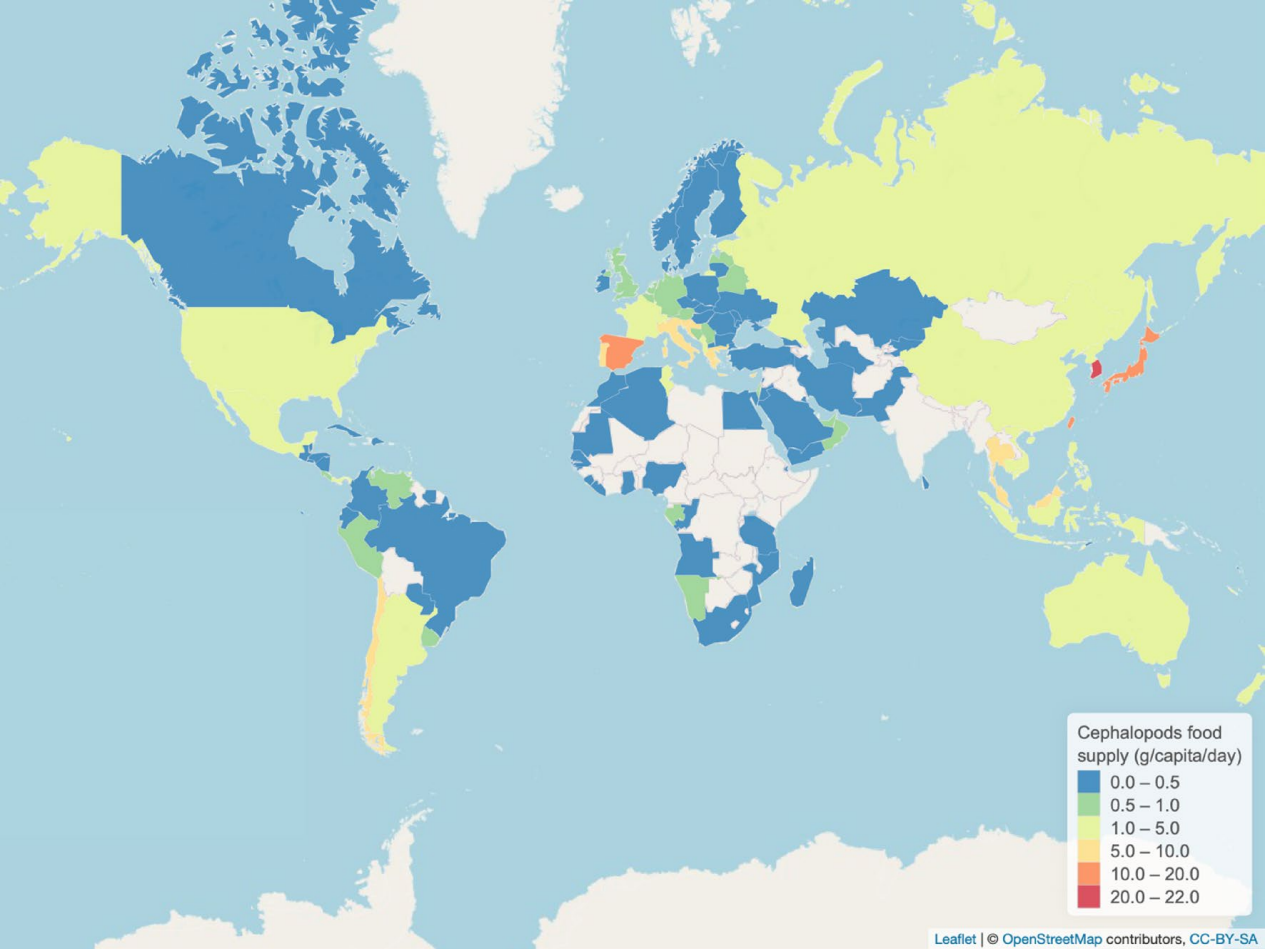
Worldwide cephalopod food supply in g/capita/day for 2013. Per capita supplies are only the average supply available for everyone in the country or territory population as a whole and do not show what is actually consumed by individuals. The figure was created with Leaflet (https://leafletjs.com)^14^ for R^12^ (https://cran.r-project.org) by using FAO cephalopod food supply data^8^ and the continents layout from OpenStreetMap (https://www.openstreetmap.org).

To decipher the cephalopod trade network, it is important to understand the dynamics of both the key supplier and the main consumers of cephalopods over time. According to the Food and Agriculture Organization^2,8^, Eastern and Southeast Asian and Southern European countries or territories had the highest per capita supply of domestic cephalopods in 2013 (Fig. 2). In 2013, the Republic of Korea, Japan, Taiwan, and Spain had the highest availability of cephalopods for local consumption, all exceeding 10 g/capita/day (Fig. 2). Although the Republic of Korea and Japan dominate cephalopod consumption today, squid consumption in some Asian countries, such as Japan, has declined since the 1980s. Conversely, in Spain, consumption of all cephalopod groups has increased in line with imports (in volume) since the 1980s, although catches by the Spanish fleet have gradually declined^2^.

Despite good knowledge on the current global state of cephalopod catches and consumption, vast information gaps exist about the major players in the cephalopod global seafood market. Faced with one of the world’s greatest challenges—how to feed more than 9 billion people by 2050 in a context of climate change, economic and financial uncertainty, and growing competition for natural resources—the international community made unprecedented commitments in September 2015, when UN Member States adopted the 2030 Agenda for Sustainable Development (UN SDG), namely UN SDG 14 (Life Below Water)^9^. As global fish stocks have been progressively overfished^2^, global cephalopod biomass has increased^10^; however, there is evidence of overexploitation of some cephalopod species. To achieve the UN SDGs, complex interactions between the ecological abundance of commercial marine species and economic trends need to be fully understood. Ecological^10,11^ and economic studies have focused on biomass and environmental changes in key cephalopod fisheries (e.g., the Patagonian shortfin squid fishery)^4^, while global patterns of cephalopod seafood markets are still unknown.

Global fisheries and trade databases have been extensively analysed to extract the main harvesting, importing, and exporting traders (countries or territories); however, less effort has been devoted to understanding international cephalopod trade flows and their characteristics, despite their scale and scope. By addressing this knowledge gap, complex network methods have the potential to analyse the global trading system in a way that reveals many new topological and dynamic features of a network of interacting elements (e.g., stakeholders, enterprises, or countries). One relevant topic in the study of world trade is to explore the role of traders as geographical entities, their influences, and the situation they occupy in the network^15–18^. The links (a.k.a., edges or arcs) between nodes (a.k.a., vertices or nodes) of the network (a.k.a., graph) represent interactions/connections between entities and can be weighted, for instance, according to the volume of commodities or money value traded between countries. The network analysis considering the weight of links may provide further information on the network structure. In a trade network, the graph represents the network itself, with each trader being a vertex or node, and the probability of connection or flow of commodities between traders being the arcs or edges. Network analysis provides insights into the system properties and identifies critical nodes with high centrality (i.e., connected to many other traders) or clusters of well-connected nodes with high potential trade flow and acting as bridges between distant world regions. Centrality is a measure that indicates the relevance of a node in a network. It should measure the 4 P's—Prestige, Prominence, imPortance and Power^19^. Each node could be important from a different point of view depending on how that “relevance” is defined. The study of centrality in network analysis is intended to identify the most important nodes in a graph given its topology.

The aim of this work is to apply network analysis to the cephalopods trade network to account for the position of the different countries within the network and reveal the main actors channelling the flow of commodities at a global scale. We explored the cephalopod trade flow globally by using 20 years of records compiled by the UN COMTRADE, the United Nations International Trade Statistics Database, freely accessible at https://comtrade.un.org/data/. The database used includes over 185,000 records, including the flow between traders weighted by volume (kg) and by monetary value (USD). By exploring different measures of network centrality, we could assess emerging patterns in what we called the Cephalopod Global Trade Network (CGTN) to identify the most relevant actors for cephalopod trade.

## Methods

In this study, we compute measures of network analysis and apply network graph visualization tools to cephalopod trade flows to understand their nature and dynamics at a planetary level. Specifically, we study the octopus, squid, and cuttlefish global trade networks where the extent of trade between a pair of countries can be treated as the link weight. Data was extracted for 252 countries or territories and 20 consecutive years (2000–2019) from the UN COMTRADE database. Data extraction was done through the COMTRADE API using the package “comtradr” v.0.2.2^20^ for the R language and environment for statistical computing version 4.0.3, released on 2020-10-10^12^. The COMTRADE API requires that searches for specific commodities are done using commodity codes. Codes used for cephalopods are listed in Table S1.

Using these codes, we conducted queries on all imports and exports reported by any trader from 2000 to 2019. The output was a database having each transaction reported by trader organized in a timestamped (year) origin–destination format, followed by the quantity of the product traded in volume (kilograms) and the value of the transaction (USD). The traded products were identified as either fresh (live, fresh, or chilled product) or elaborated (frozen, dry, salted, in brine) for (1) octopus and for (2) cuttlefish and squid. These two categories are predefined in the COMTRADE database following the Harmonized System Nomenclature ("HS") and cannot be disaggregated by species or other taxonomic groups. The HS is an international customs terminology for the classification of goods that is currently applied by more than 200 traders around the world. Data were processed and analysed to rank the top five exporters, importers and trade flows regarding volume or monetary value in four 5-year periods (i.e., 2000–2004; 2005–2009; 2010–2014 and 2015–2019).

We used network analysis to establish sound theoretical connections between traders involved in the CGTN and analyse the emergent structure of the underlying network of trade connections. We first constructed a directed graph weighted by monetary value (USD) or volume (kg). We considered these two measures to identify potential different patterns in the trade network driven by monetary or by volume transactions. Each node in the graph is identified as a country or territory involved in a trade transaction. However, since not all traders shared trade relationships, the number of nodes was always less than the 230 traders originally identified in the database.

The relationship between each pair of nodes was identified in the network with a link (edge) and the nature of the trade operation (export or import) is determined by the directionality of the link. Therefore, the directionality was denoted with an arrow pointing to the importing country or territory. Each edge in the graph was weighted by the total monetary value or volume involved in all transactions between two nodes over time. Therefore, the size of the nodes represents the relevance of the traders in the global trade network according to the sum of the weights of the edges, in monetary value or volume, flowing from or to each trader (i.e., the node strength or the weighted degree of the nodes). The edges represent the flow of trade, the width of the edge represents the quantity of commodity traded between two nodes. Multi-annual data were normalised using min–max normalization to indicate relevance rather than gross values. In min–max normalization, for every feature, the minimum value of that feature gets transformed into a 0, the maximum value gets transformed into a 1, and every other value gets transformed into a decimal between 0 and 1. Finally, for a better understanding of the trade relations between the different countries and territories, the nodes were geolocated on a world map.

To identify emerging properties within the CGTN, we calculated different centrality measures considering trade links generated by aggregate gross export flows (in either monetary value or volume). For each pair of trading countries, the number of transactions occurring within a year were obtained and summed. In this way, for each pair of traders a single value per year was estimated. For specific periods (e.g., a 5-year period), the annual amounts traded by each pair of traders were summed. Centrality measures are useful to determine the relative importance of nodes and edges within the overall network^21^. In networks consisting of several nodes (e.g., social networks, Marine Protected Area networks, food webs, metapopulation connectivity, etc.), some nodes play a decisive role in facilitating many network connections^22–26^. Such nodes are central in network organization and are often identified by a range of metrics known as centrality measures. Here, we calculated 9 measures of centrality for the CGTN: Degree, In-degree, Out-degree, Strength, In-strength; Out-strength; Closeness; Betweenness; and PageRank. We selected these centrality measures as those metrics potentially useful in trade network studies. They are a product of a first screening that included all existing measures of centrality, identified from a review of the existing literature. A full description of each centrality measure selected, its scope, and market interpretation, is provided in Table S2. Note that Degree of a node is the number of edges that arrive at that node. In a directed graph the degree is usually divided into the In-degree and the Out-degree (whose sum is the degree of the node). Out-strength and In-strength correspond to the weighted Out-degree and weighted In-degree of the node, respectively, and Strength (weighted Degree) corresponds to the sum of In-strength and Out-strength.

Hierarchical clustering of agglomerations using the Ward's clustering method was used to produce groups of traders that minimize within-group dispersion at each binary fusion. A priori statistical significance of the clusters was tested using the similarity profile (permutations = 999, number of expected clusters = 1000) of the members of the identified density clusters.

All analyses were performed using the R language and environment for statistical computing^12^. Network graph analyses were performed using R package “igraph” v.1.2.5^27^. Hierarchical clustering analyses were performed using the package “flashClust” v.1.01-2^28^. Network visualisations were made with R packages: “ggplot2” v.3.2.1, “ggmap” v.3.0.0 and “ggraph” v.2.0.0^13,29,30^. All the databases, the codes for the analyses and the scripts to produce the visual representation of the networks are publicly available on GitHub^31^.

## Results

### Trends in cephalopods trade

Since 2000, trade in fresh octopus has been constantly dominated by the flow from China to Korea, followed by Vietnam to Japan, Portugal to Spain and Spain to Italy. However, there has been a marked decrease in the traded volume and monetary value over time, with a 50% reduction in the top 5 traders (Tables S3–S5).

Over the last 20 years, fresh octopus exports have been strongly dominated by China, followed by Spain, Vietnam, Portugal, and France, and recently by Morocco and Thailand (Table S3). While Vietnam was the most important exporter in the first period (2000–2005), it was not within the top 5 traders in the last 5 years. Imports have been dominated by Korea, Italy, and Portugal, with no notable changes in the whole period (Table S4). Regarding trade of processed octopus, the largest transactions have been performed from Morocco to Spain, Morocco to Japan, Mauritania to Japan (and more recently also to Spain) and China to Korea (Table S5). Since 2000, exports of processed products have been dominated by Morocco, Mauritania, China, Spain, and Vietnam (Table S3), while imports have been led by Japan, Spain, Italy, Korea, and the United States (Table S4).

Trade in volume of fresh cuttlefish and squid includes fewer clear relationships over time, such as transactions from Malaysia to Singapore (2000–2004 and 2005–2009); from Myanmar to Thailand (2005–2009 and 2015–2019); and from Yemen to Vietnam (2010–2015) (Tables S6-S8). For the first 5 years, exports of fresh commodities were dominated by Vietnam. However, since 2005, India, Spain and France have increased their exports in both monetary value and volume, displacing Vietnam from the top rank (Table S6). The main importing traders were Spain and Italy. Although China was important in the first decade, it was replaced by Vietnam in the last decade (Table S7). The trade of elaborated cuttlefish and squid products was dominated by monetary value flow from Thailand to Japan and from Malvinas/Falkland Islands to Spain in the first decade, while in the last decade the flow from China to other traders (Japan, the USA, and Thailand) gained relevance. However, the volume follows a different pattern, with flow from the Malvinas/Falkland Islands to Spain and from Korea to China in the first decade, while in the last decade, flows from Peru to China and from China to Thailand were important (Table S8). A disparity exists between the top five traders in terms of flow of monetary value and volume in the first 15 years; but in the last 5 years the top positions are constantly represented by, China, Peru, India, and Spain. Italy, Japan, China, and the USA are important importers in terms of monetary value and volume, although in the last decade Thailand has increased its importance, replacing the USA in the top 5 in the last 5 years (Table S7).

**Figure 3:**
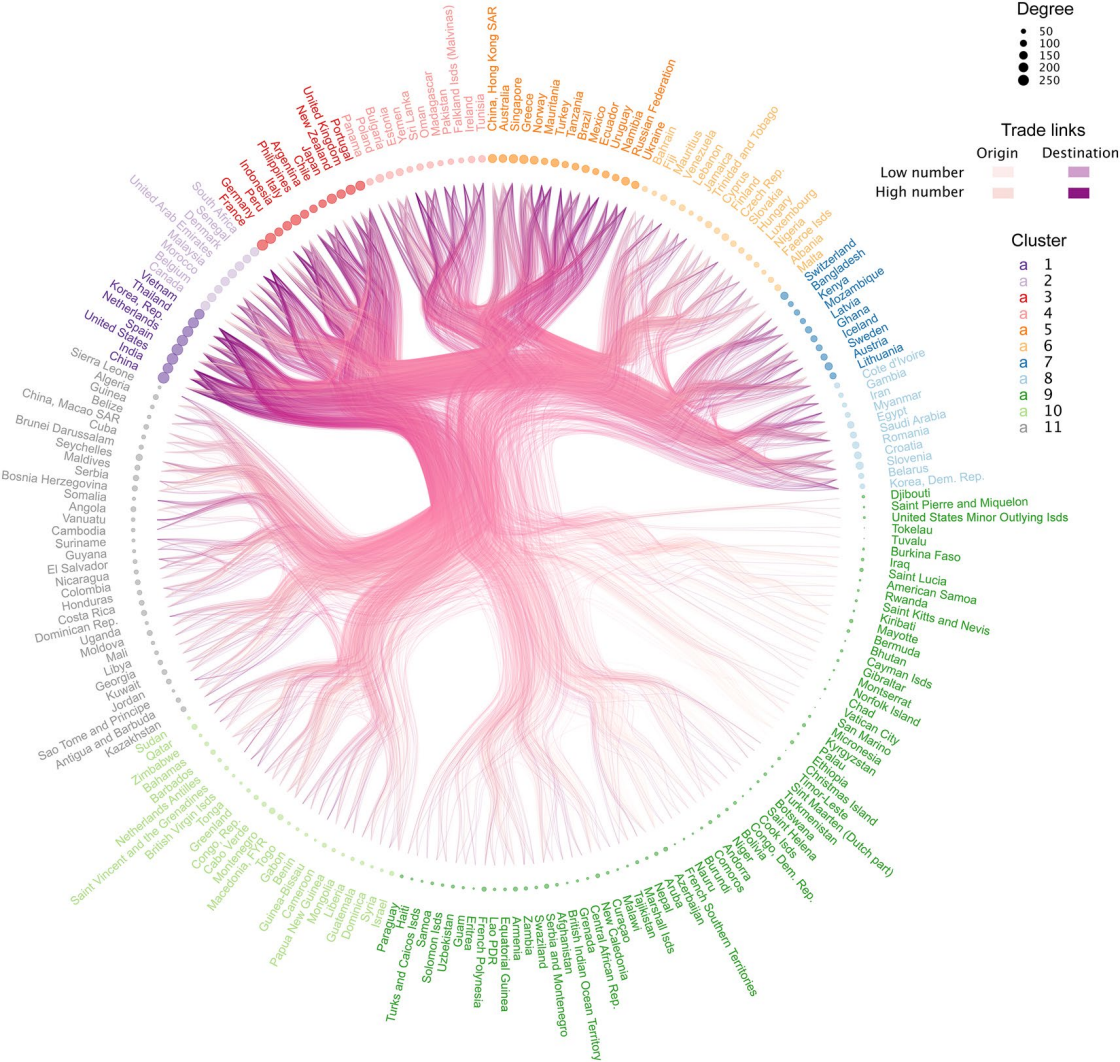
The Cephalopod Global Trade Network. The top 220 traders of the CGTN as nodes (circles) and their trade links as lines. The colour and the size of the nodes represent, respectively, the cluster membership and relative importance of the trader in the CGTN, estimated from the number of trade links with other traders (i.e., degree). The colour of the edges represents the origin, destination and the proportion of trade links for all years between each pair of traders. The clusters were made using Ward's method. The figure was created with R^12^ (https://cran.r-project.org) packages: “ggraph” v.2.0.0^30^ (https://ggraph.data-imaginist.com) and “ggtree” v3.0.2^32^ (https://guangchuangyu.github.io/ggtree-book/chapter-ggtree.html).

The CGTN involved 220 traders (countries or territories) from around the world with exports greater than or equal to 500 kg between 2000 and 2019 (Fig. 3). The remaining 32 traders either did not report exports or their exports were below 500 kg. The most important cluster of traders was composed by 8 countries that dominate the cephalopod global markets in Asia (China, India, Republic of Korea, Thailand, Vietnam), Europe (the Netherlands, Spain) and the USA. The second and third most relevant clusters were composed of 8 and 12 traders, respectively. These two clusters involve 9 developed countries (Belgium, Canada, Denmark, France, Germany, Italy, Japan, United Kingdom and Portugal) and 11 developing countries (Morocco, Malaysia, UAE, Senegal, South Africa, Peru, Indonesia, Philippines, Argentina, Chile and New Zealand). Some of these traders have the most productive cephalopod fisheries in the world (e.g., Patagonian shortfin squid in the Southwest Atlantic Ocean and Patagonian squid in the Southeastern Pacific Ocean).

### Octopus trade network

#### Live, fresh, or chilled octopus

**Figure 4:**
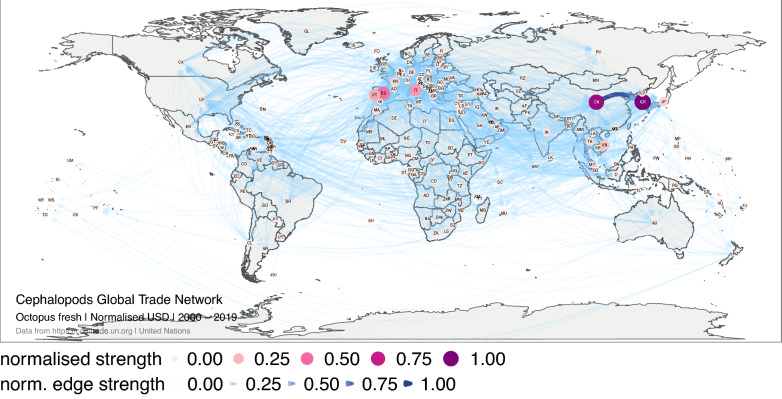
Global trade network for octopus live, fresh or chilled between 1 January 2000, and 31 December 2019 in monetary value (USD). The numbers correspond to the normalised strength for the monetary value. Each node represents a trader, and each edge represents the export–import relationship between two traders. The size and colour of the node represent the relative importance of the trader in the network in terms of its strength. The width and colour of the edge represent the relative importance of the relationship between two traders in terms of their edge strength. The figure was created with R^12^ (https://cran.r-project.org) packages: “ggplot2” v.3.2.1^13^ (https://ggplot2.tidyverse.org), “ggmap” v.3.0.0^29^ (https://github.com/dkahle/ggmap) and “ggraph” v.2.0.0^30^ (https://ggraph.data-imaginist.com).

The normalised strength (Fig. 4) revealed the importance of China and Republic of Korea in the trade of fresh octopus in monetary value, with high importance of flows between these two traders over time (Supplementary Fig. S1). Other relevant traders over time were Spain, Portugal and Italy, in Europe; and Vietnam and Japan, in Asia from 2000 to 2004 (Supplementary Fig. S1). The network based in volume showed similar results.

The Betweenness identified important actors facilitating flow through the network. For fresh octopus, the most relevant traders in the last two decades were Spain, France, and Italy, followed by Thailand, Portugal and the USA. Again, no major differences exist between the monetary value and volume networks. However, the ranking of traders changed over time, with Italy replacing Spain in the first place during the period 2005–2009, and then Spain consolidating again in the first place in the following periods. Also, in the last 20 years there were many changes in Asia, with Vietnam losing and Korea gaining prominence over time (Supplementary Fig. S2).

**Figure 5:**
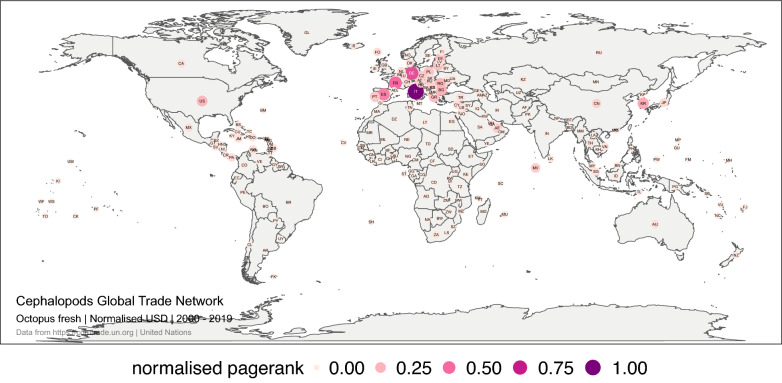
Global trade network for octopus live, fresh or chilled between 1 January 2000, and 31 December 2019 in monetary value (USD). The numbers correspond to the normalised PageRank for the monetary value. Each node represents a trader. The size and colour of the node represent the relative importance of the trader in the network in terms of its PageRank. The figure was created with R^12^ (https://cran.r-project.org) packages: “ggplot2” v.3.2.1^13^ (https://ggplot2.tidyverse.org), “ggmap” v.3.0.0^29^ (https://github.com/dkahle/ggmap) and “ggraph” v.2.0.0^30^ (https://ggraph.data-imaginist.com).

In a global trade network, there are countries that are essential to the network structure because they are connected to other countries critical to the network and those critical countries, in turn, have no other significant connections. PageRank is a centrality measure that identifies these important countries, resulting from an iterative algorithm that assigns higher values to countries with a greater number of import connections with other countries that move large quantities of goods or money^33^. In the last 20 years, Italy, Germany France and Spain have occupied those central positions in the global trade network of live, fresh or chilled octopus. Their dominance has not changed over the four 5-year periods analysed (Fig. 5).

#### Elaborated octopus

The normalised strength revealed a diversified trade network for elaborated octopus products. This network has remained relatively stable over the last 20 years, with some exceptions. In the 2000s, there was an intense flow of exports from North Africa (i.e., Morocco and Mauritania) to Japan, although the most important flow was from Morocco to Spain. In the 2010s, Mauritania changed its preferential partner and exported large quantities of elaborated octopus to Spain, with the latter gaining dominance. In this last decade, flows from China and Vietnam to the Republic of Korea also became important. Other relevant actors were distributed globally (e.g., Italy, Portugal, Senegal, the USA). However, the most important routes showed a common pattern: the origin was in developing countries or territories (that emerged as producers) while developed countries showed a high and stable consumer demand (Supplementary Fig. S3). The network based on volume was highly similar to the monetary value network. However, Italy, China, Korea, Vietnam, and the USA reduced their importance compared to the top-ranked traders (i.e., Spain, Japan, and Morocco). The most important routes of the volume network were from China to Korea; Morocco to Spain; Morocco and Mauritania to Japan and Vietnam to Korea.

The Betweenness measure highlighted the role of Spain as a facilitating actor in the trade network of elaborated octopus, followed by Italy, China, and the USA. These countries have maintained their importance as structurers of the world trade network in elaborated octopus over the past 20 years. Similarly, the routes from Italy to Spain, and from Spain to China and the USA emerged as relevant in the network structure, with a special mention to the route between Japan and China in the last 5-year period (Supplementary Fig. S4). There are no major differences between the most central traders in this network and the volume-based one.

PageRank revealed the importance of Spain and Italy as leading traders in the elaborated octopus market. These two countries concentrated a large number of import relationships, which also concentrated a large monetary and volume flow. This importance has been maintained over time. Other reference actors were Greece, Japan, the USA and Portugal, in the first decade; and the USA, Portugal, Greece and Korea in the second decade. Note how in the second decade, Greece and Japan lost relevance, while Northern European countries and the Republic of Korea gained relevance (Supplementary Fig. S5).

### Squid and cuttlefish trade network

#### Live, fresh, or chilled squid and cuttlefish

**Figure 6:**
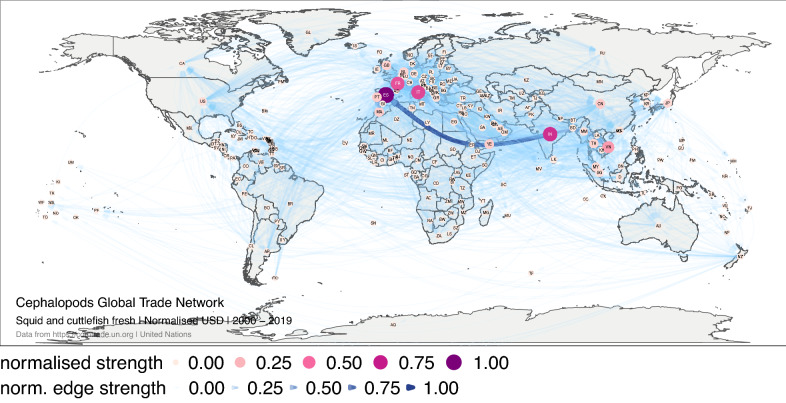
Global trade network for squid and cuttlefish live, fresh or chilled between 1 January 2000, and 31 December 2019 in monetary value (USD). The numbers correspond to the normalised strength for the monetary value. Each node represents a trader, and each edge represents the export–import relationship between two traders. The size and colour of the node represent the relative importance of the trader in the network in terms of its strength. The width and colour of the edge represent the relative importance of the relationship between two traders in terms of their edge strength. The figure was created with R^12^ (https://cran.r-project.org) packages: “ggplot2” v.3.2.1^13^ (https://ggplot2.tidyverse.org), “ggmap” v.3.0.0^29^ (https://github.com/dkahle/ggmap) and “ggraph” v.2.0.0^30^ (https://ggraph.data-imaginist.com).

The normalised strength revealed the importance of Spain, France, Italy, and India in the trade network of fresh squid and cuttlefish products, especially the route between east Asia and Spain (Fig. 6). The volume-based network is highly similar to the monetary value network. Over the four 5-year periods analysed, Vietnam and Japan have gradually lost relative importance in the network (Supplementary Fig. S6).

For fresh squid and cuttlefish, Betweenness identified Spain as the most important structurer of the network, in both monetary value and volume, over the time. Europe emerged as a major region structuring the global trade network. While in Asia, the exchange of countries with higher betweenness over time evidences the strong struggle for control of trade in the region and the more fragile sub-networks. In the monetary network, in the 2000s the bridge between the United Kingdom and Korea stood out, while in the 2010s the Europe-Asia bridge was established between Spain and India. In the volume-based network, it is noteworthy that in the 2010s there were many critical routes for the stability of the network, even in the Europe–Asia connection. Note, for example how the link between Netherlands and Myanmar stand out (Supplementary Fig. S7).

**Figure 7:**
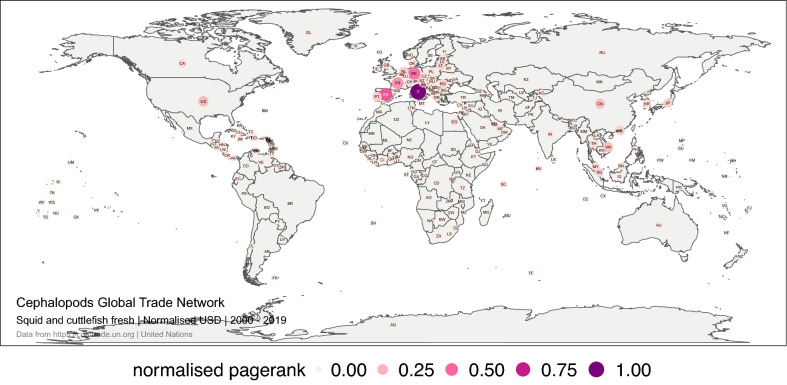
Global trade network for squid and cuttlefish live, fresh or chilled between 1 January 2000, and 31 December 2019 in monetary value (USD). The numbers correspond to the normalised PageRank for the monetary value. Each node represents a trader. The size and colour of the node represent the relative importance of the trader in the network in terms of its PageRank. The figure was created with R^12^ (https://cran.r-project.org) packages: “ggplot2” v.3.2.1^13^ (https://ggplot2.tidyverse.org), “ggmap” v.3.0.0^29^ (https://github.com/dkahle/ggmap) and “ggraph” v.2.0.0^30^ (https://ggraph.data-imaginist.com).

PageRank revealed the importance of Italy, Spain, Germany and France as leading traders in the fresh squid and cuttlefish market. In the last 20 years, Europe concentrated the largest number of import relationships, which also concentrated a large monetary and volume flow. Europe leadership has been maintained over time (Fig. 7).

#### Elaborated squid and cuttlefish

The trade networks based on monetary value and volume for elaborated squid and cuttlefish emerge as global and complex, where several far distant traders have relevant roles in the import/export network (Supplementary Fig. S8). Although the most important nodes in the volume-based network reflected important nodes in the monetary value network, the strengths of the links, i.e., the flow of value and volume, did not. For example, in the volume-based network, Peru exported the largest quantities of squid and cuttlefish to China (Supplementary Fig. S8b,d), but the flow of money for these transactions was less important (Supplementary Fig. S8a,c).

The betweenness centrality metric (based both on monetary value and volume) showed the importance of China, the USA, and Spain (followed by Italy, Korea, and Thailand) as facilitators in the elaborated goods trade network (Supplementary Fig. S9). While the main important bridges in volume transactions were between Italy and Spain, Spain and China, and China and the USA (Supplementary Fig. S9b,d), the main monetary bridges were from the USA to China, followed by the routes from Spain to the USA and from Italy to Spain (Supplementary Fig. S9a,c). The key traders structuring the network were the same, but they follow different directions.

**Figure 8:**
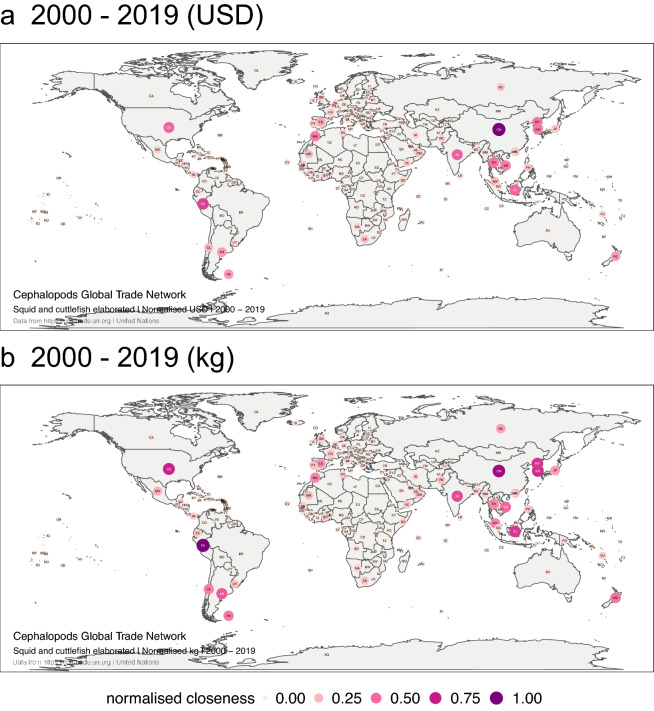
Global trade network for squid and cuttlefish elaborated between 1 January 2000, and 31 December 2019 in monetary value (USD) above, and volume (kg) below. The numbers correspond to the normalised closeness for the monetary value (USD) and volume (kg) traded, respectively. Each node represents a trader. The size and colour of the node represent the relative importance of the trader in the network in terms of its closeness. The figure was created with R^12^ (https://cran.r-project.org) packages: “ggplot2” v.3.2.1^13^ (https://ggplot2.tidyverse.org), “ggmap” v.3.0.0^29^ (https://github.com/dkahle/ggmap) and “ggraph” v.2.0.0^30^ (https://ggraph.data-imaginist.com).

Closeness centrality highlighted the main actors in a regional context (Fig. 8). In both the monetary value and volume-based networks, China, North and South Korea, India, Indonesia, Thailand, and Vietnam form a strong trade network for squid and cuttlefish elaborated in Asia. Key players include South America (Peru, Argentina, Chile, the Malvinas/Falkland Islands); the USA; the Mediterranean (Morocco, Spain); Africa (South Africa, Mauritania); and the West Pacific region (New Zealand, Japan). Note how the highest values of closeness were slightly different in the money-based network (Fig. 8a) and in the volume-based network (Fig. 8b), mainly for those countries that are historically large producers of elaborated squid and cuttlefish (e.g., Peru and Argentina).

PageRank revealed the importance of Greece, Spain, China, USA and Japan as leading traders in the global elaborated squid and cuttlefish market for the first 10-years (Supplementary Fig. S10a). Globally, in the last decade, China, the USA and Japan have declined in importance, while Europe has consolidated its importance. However, Spain's relevance has declined over the last decade while Germany's has increased (Supplementary Fig. S10b).

## Discussion

Although trade involving cephalopods increased its contribution to the global seafood market in monetary terms (USD), as well as in volume, from 2000 to the mid-2010s, there has been limited research to describe its global scope and scale. There is no specific regulation, nor have monitoring systems been implemented, to study the traceability of cephalopods at the international level.

In European waters, the catch of cephalopods in large-scale fisheries is virtually unregulated. Since cephalopods are often not the target species, their catches are only indirectly controlled, for example through restrictions on the types of fishing gear that can be used and catch quotas set for non-cephalopod species. In artisanal cephalopod fisheries, especially in southern Europe, regulatory restrictions on fishing activity are numerous, but few regulations are aimed at maintaining the status of cephalopod stocks, and these regulations are not always enforced, resulting in suspected high levels of illegal, unreported and unregulated (IUU) fishing ^34^.

Sanctions on international trade agreements, though hard to implement, can be a persuasive tool to discourage unsustainable practices such as IUU fishing^35^. For example, the European Commission has repeatedly sanctioned seafood imports from Southeast Asian traders related to IUU fishing. Countries like Vietnam joined the Association of Southeast Asian Nations (ASEAN) to combat such practices and to benefit from a new Free Trade Agreement with the EU with 99% of tariffs eliminated, including those for octopus and squid products^36,37^. Other impediments to trade tend to include weak transportation links, inefficient customs clearance, bureaucracy, and red tape^38^. The European Union’s Rapid Alert System for Food and Feed (RASFF) detects risks to health in consignments of food imports into the EU, such as the presence of heavy metals or breaches of the cold chain which can lead to border rejection^39^. In this context, trade control is a powerful tool to regulate unsustainable practices of the largely unregulated cephalopod fisheries. We provide the most comprehensive description of the legal trade in cephalopods to understand the routes and key world players with a methodology that, systematically applied, could contribute to achieving transparency and traceability of seafood markets.

In the context of global trade, the ability of a given trader to connect with other traders that demand products from it, thus creating a flow of goods, is often referred to as connectivity. Therefore, from an economic perspective, the main concern is the role of each trader and its influence on the trade network, which is closer to the term "centrality" from a network analysis perspective. Many economic studies use well-known centrality measures, but do not identify them as such^40^. In this study, we have identified the best-connected traders according to different measures of centrality weighted by monetary value or volume. A PageRank-based approach offers a ranking that is independent of the amount of trade from a given country. Countries with a high PageRank value are large importers from many exporters; it is not the volume that is decisive but how extensive and diversified their network of trade contacts is. These countries can act as important hubs in world trade. It is not surprising that the highest PageRank values are held by European countries in most of the analysed networks. Another centrality measure, Betweenness, reveals the modulators that connect clusters of traders. The elimination of a modulator can fragment the trading network. For example, in the elaborated octopus trading network, Spain, Italy and China are key network modulators.

Our analysis reveals that traders with the highest import and export rates in many cases are not the most important gateways. In the case of fresh octopus, China and Korea were the largest capital players (highest Strength), but Italy was the trader with the more diversified market network (highest PageRank). However, three European countries (Spain, Italy, and France), with the highest Betweenness, played a critical role for connectivity at the international level. They acted as bridges between different clusters of traders, and thus allow traders from different clusters to be connected not only with their neighbours, but also with traders from other clusters. The commercial routes for fresh octopus have been strongly dominated by four trade flows concentrated in China and Southern Europe: China to Korea, Portugal to Spain and Spain to Italy and Portugal. These countries are characterised by high consumption of octopus (e.g., Korea, Spain, Italy), or by the size and scale of its production and transformation, and with the capacity to shift global seafood markets (e.g., China). Note the particularity of the bi-directional route: Portugal-Spain-Portugal, as Portugal exported fresh octopus products to Spain and Spain to Portugal. Yet, whether these exports corresponded to different catches or to redistribution of the same product remains unclear. These are the principal routes, and although the quantities of products traded through these routes have markedly decreased in recent years, the monetary value has increased.

In monetary terms, the flow of capital has changed from being highly diversified, with several traders in the top 5 in the first decade of the 2000s, to almost total dominance by China in the last decade. These patterns illustrate the increasing complexity of the international market for cephalopods, where efficient processes for logistics and meeting standards and regulatory requirements are critical to participation in the global value chain (GVC) while low unit labour costs are critical for competitiveness^38^.

Elaborated octopus is principally traded from China to Korea. However, the routes are more diverse and variable over time, with important routes including Morocco to Spain, Italy to Japan, Mauritania to Japan, and Vietnam to Korea. The list of top exporting traders for processed octopus is also diverse including countries like Vietnam, Mauritania, and Spain. Note that the trade routes for live octopus are characterised by proximity between traders (e.g., Spain, France, Italy, Portugal), while processed octopus trade routes are longer (e.g., Morocco to Japan or Mauritania to Japan). This rule is not true for fresh squid and cuttlefish, with a trade from India to Europe being one of the important routes.

Proximity and agglomeration benefits are important. For example, in the elaborated squid and cuttlefish trade network, although numerous Asian and European traders have many connections and a considerable flow of money and volume with other traders, South American producers (i.e., Peru, Chile, Argentina and Malvinas/Falkland Is.) occupy a central strategic position with high leverage among its proximate partners (high closeness).

Geography is important for developing countries to take part in cephalopod trade. Developing countries tend to trade with the hub that is geographically closest, with large firms tending to be involved in global production networks while small firms trade within the region^38^. See for example the case of Peru, which imports a large quantity of fresh octopus from Chile for further processing or reallocation, a typical example of a trade relationship mediated by geographical and cultural proximity.

However, global trade networks are complex and geographic proximity does not imply trade proximity. For example, Morocco and Mauritania are geographically close but have a much lower trade flow of processed octopus between them compared to the trade each has with several Southeast Asian traders. On the other hand, Peru (in monetary terms) and Indonesia (in volume terms), are aggregators or accumulators of fresh octopus imported from a few trading partners and then exported to many other traders, through networks involving numerous connections. In the other direction, there are traders, such as South Africa in the elaborated squid network, that act as aggregators, but in this case importing (enormous quantities in USD) from many different traders and then exporting to a few destinations.

All the above situations may be due to multiple or interrelated factors such as high GDP, foreign direct investment, the presence of trade agreements, economic complementarity, and historical and cultural ties that make a country or territory the most important trading partner for a single country or group of countries^40^. The flow of elaborated squid and cuttlefish in volume presents some interesting particularities that, as mentioned, could be affected by external factors. For example, the strong Malvinas/Falkland Islands-Spain relationship and, in the last decade, Peru-China. This scenario might change as Brexit-associated economic risks include the adoption of new taxes for cephalopod exports for Spanish fishing vessels which have been operating in Malvinas/Falkland waters over the last three decades.

We identified the second most relevant cluster in the CGTN as being composed of eight developing countries (Argentina, Chile, Malaysia, Morocco, Philippines, Senegal, South Africa, UAE), some of which host the most productive cephalopod fisheries in the world. Our results also identified that elaborated octopus products tend to move from developing to developed countries. These findings reflect the global North’s increasing importance as a net importer of natural resources from the South, showing a high specialization on a growing demand for cephalopods.

Simultaneously, these results also cause traders in the South to place greater economic importance on resource intensive primary sectors and taking on a greater environmental burden as a result^41^. Some developing countries have trade deficits due to exporting more fisheries resources to developed countries than they import. There is ongoing debate about whether this is beneficial or detrimental to the exporting traders in terms of loss of access to the exported foods compared with increased purchasing power from income generated from those exports^42^. The flow of cephalopod products may contribute to food distribution equity by improving access to nutrient rich foods across countries or territories and socio-economic groups^43^.

Finally, there are some key challenges that need to be addressed in order to improve our scientific understanding on the interlinkages between cephalopods fisheries and trade in the next years. A growing demand for cephalopods can lead to increased exploitation of stocks with implications for their environmental, social, and economic sustainability, in particular, in developing countries where corruption and IUU fishing practices are often the logical response to a lack of effective policy and regulatory frameworks^44,45^. Furthermore, the energy cost of fishing and transporting cephalopods through the complex trade links as well as the carbon sink prevented by the removal of cephalopods we have described from the oceans^46^, must also be considered in terms of sustainable cephalopods exploitation and carbon emissions.

Another challenge to be considered is that cephalopods are one of the fastest growing products in terms of market share in the global seafood trade, rising from 13% in 2000 to 16% at their peak in monetary terms between 2014 and 2016, and from 29% in volume terms in 2000 to 30% in 2014. However, in the last 3 years they have reached their lowest global seafood market share for the last two decades, at 5% in both monetary value and volume. On average, the price of fresh octopus was ca. 2.2 USD/kg in 2000, and by 2018, it had increased fivefold to ca. 11.6 USD/kg. Similarly, but at a lower rate, the traded quantities of elaborated octopus have decreased in recent years and the monetary value of these transactions has increased. On average, the trade of elaborated products has increased from 2.6 USD/kg in 2000 to 10.2 USD/kg in 2019. These results suggest that trade for live, fresh or chilled octopus, is better positioned in the market, either due to growing consumer interest or to a shift towards healthier consumption habits. There is a growing interest among chefs and gastroscientists to promote novel uses of cephalopods to replace meat from land-animal production^47^.

The complexity of the cephalopods trade flows we have described along with variations in (or lack of) labelling systems and official lists of seafood trade names in different countries or territories can make it difficult to accurately identify the origin of raw material used in cephalopod products, especially in processed preparations where potentially identifiable anatomical features have been removed^48^. Lack of traceability measures creates opportunities for exploitation through product mislabelling or substitution with species of lower commercial value, as well as abusive practices such as the addition of water to artificially increase product weight and, ultimately, the price^48–50^. Mislabelling can have significant impacts on efforts to sustainably manage associated fishers^51^. There are several ways of communicating to consumers about the environmental sustainability of seafood products and fishing activities, mostly done through labelling, certification and ratings programs. The most rigorous and credible ones have been recognized by international initiatives, such as the Global Sustainable Seafood Initiative (GSSI) and the Certification and Ratings (Cert&Rat) Collaboration, that analyse the alignment of these programs with the FAO Code of Conduct for Responsible Fisheries and the FAO Guidelines for the Ecolabelling of Fish and Fishery Products from Marine Capture Fisheries.

## Conclusions

Our findings show that the Cephalopod Global Trade Network involves 220 traders around the globe, involving 11 clusters of traders based on the volume and monetary value of cephalopod trade and the number of transactions between 2000 and 2019. The most important cluster is composed by only 8 traders which dominate cephalopod seafood markets located in Asia (China, India, Republic of Korea, Thailand, and Vietnam), Europe (Netherlands and Spain) and the USA.

China and the Republic of Korea have dominated the live, fresh, or chilled octopus market in the last 20 years. Vietnam and Japan had an important position as traders from 2000 to 2005, after China and the Republic of Korea. However, from 2005 to 2019, their relevance has been decreasing, which has favoured the dominance of some European and Mediterranean countries (Spain, Portugal, Italy and Morocco). The commercial routes of live, fresh, or chilled octopus have been strongly dominated by four trade flows concentrated in China and Southern Europe (i.e., China to Korea, Portugal to Spain and Spain to Italy and Portugal). Traders with a higher closeness have a high probability of exporting to the nearest neighbouring trading partners (not always geographically close), and it also identifies key traders in the regional context. The traders with the highest closeness are China, Spain, Portugal, and Vietnam. Well-connected traders have a high flow of imports and exports with traders that also have a high flow of imports and exports; while traders that have multiple trade routes with strong relationships with buyer traders prefer them over other traders in the network.

These results suggest that trade in live, fresh, or chilled octopus is better positioned in the market, either due to growing consumer interest or a shift towards healthier consumption habits. The processed octopus is also principally traded from China to Korea. However, the routes are more diverse and variable over time, with important routes including Morocco to Spain, Italy and Japan, Mauritania to Japan, and Vietnam to Korea. Trade routes for fresh squid and cuttlefish have been highly concentrated by the largest monetary transactions from India to Spain, losing relevance in the last 5 years, when the flow of capital has been greater between European traders (e.g., from Spain and France to Italy and from France to Spain).

Our findings identify the traders that act as major trade actors, modulators, intermediaries, accumulators, the best connected, the flow routes and the possible weaknesses of the global cephalopod trade network. This work provides essential input to advance towards transparent and sustainable cephalopod world trade. Given the increasing scale and speed of the cephalopod industry activity, we conclude that the industry has truly global effects today.

Finally, we highlight a few considerations when interpreting the results. First, our research does not include illegal, unreported, and unregulated (IUU) trade and discards data. Second, the level of data disaggregation for all categories of species is not always the most accurate. Third, we only focus on the trade dimension of the cephalopod industry, but global cephalopod trade could mask the constraints of marine ecosystems and thus allow actors through the value chain to ignore them by enabling substitution of input sources, or even sequential (over)exploitation^52^.

## Supplementary Information

Supplementary Figures.

Supplementary Tables.

## Data Availability

The Supplementary tables and figures can be found under the 10.6084/m9.figshare.14987001 in the public repository Figshare. To further facilitate exploration and viewing of Cehalopods Global Trade Networks by users from outside the academy or with basic technical knowledge, we have launched a fully operational web application at https://aospina.shinyapps.io/CGTN_app/.
