# Publisher Correction: A network analysis of global cephalopod trade

**DOI:** 10.1038/s41598-022-06289-2

**Published:** 2022-02-07

**Authors:** Andres Ospina-Alvarez, Silvia de Juan, Pablo Pita, Gillian Barbara Ainsworth, Fábio L. Matos, Cristina Pita, Sebastián Villasante

**Affiliations:** 1 Mediterranean Institute for Advanced Studies, IMEDEA (UIB-CSIC), C/Miquel Marques 21, CP 07190 Esporles, Balearic Islands Spain; 2 Institute of Marine Sciences ICM (CSIC), Passeig Maritim de la Barceloneta 37, CP 08003 Barcelona, Spain; 3 CRETUS Department of Applied Economics, University of Santiago de Compostela, Campus Sur, Santiago de Compostela, A Coruña, Spain; 4 Faculty of Business Administration and Management, University of Santiago de Compostela, Santiago de Compostela, A Coruña, Spain; 5 Department of Environment and Planning, CESAM-Centre for Environmental and Marine Studies, University of Aveiro, Aveiro, Portugal; 6 International Institute for Environment and Development (IIED), London, UK

Correction to: *Scientific Reports* 10.1038/s41598-021-03777-9, published online 10 January 2022

The original version of this Article contained typographical errors.

In Figure 4 and Figure 6 the complexity of the cephalopod trade network did not display correctly.

The original Figure 4 and Figure 6 and their accompanying legends appear below.

**Figure 4:**
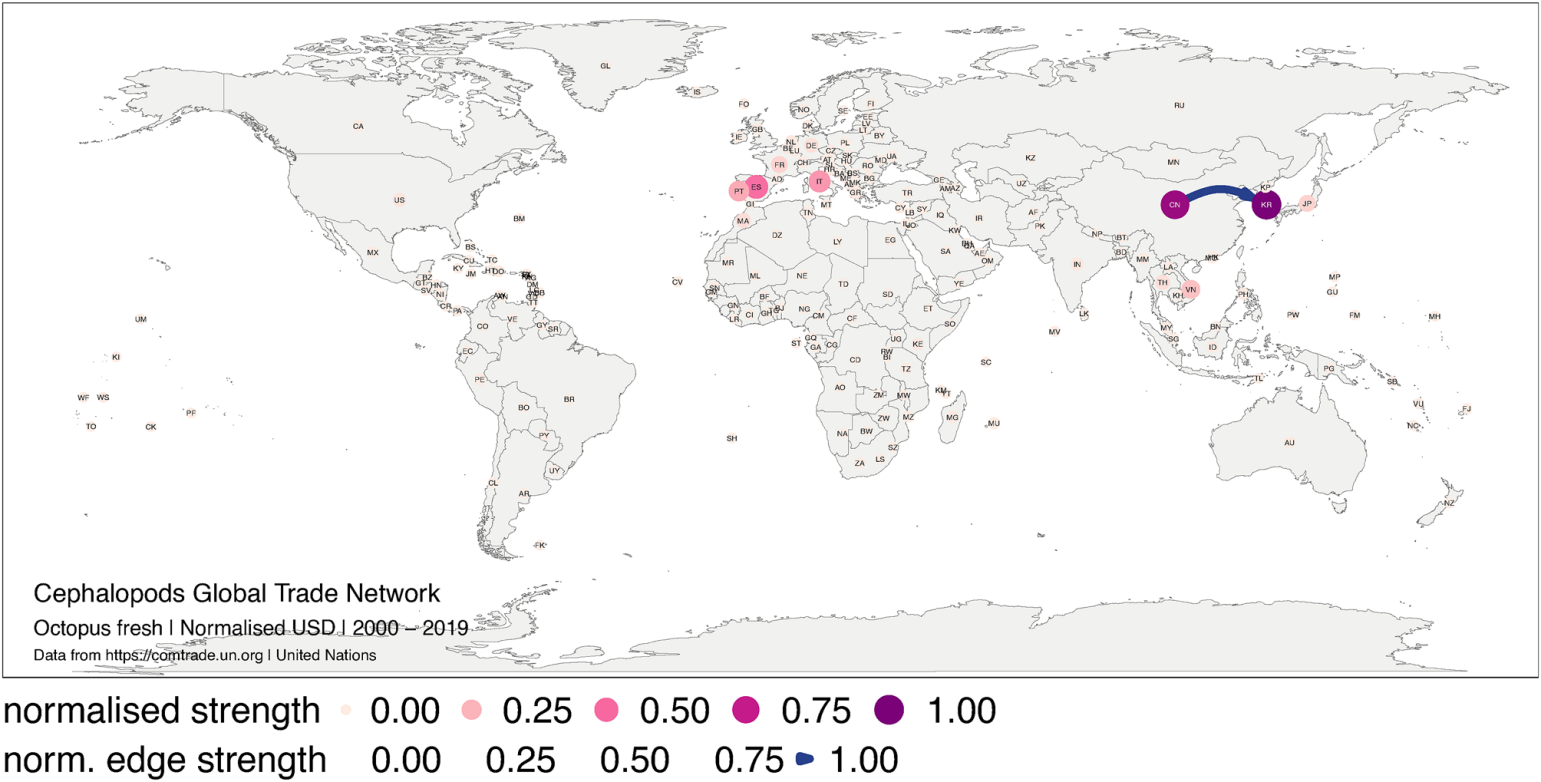
Global trade network for octopus live, fresh or chilled between 1 January 2000, and 31 December 2019 in monetary value (USD). The numbers correspond to the normalised strength for the monetary value. Each node represents a trader, and each edge represents the export–import relationship between two traders. The size and colour of the node represent the relative importance of the trader in the network in terms of its strength. The width and colour of the edge represent the relative importance of the relationship between two traders in terms of their edge strength. The figure was created with R^12^ (https://cran.r-project.org) packages: “ggplot2” v.3.2.1^13^ (https://ggplot2.tidyverse.org), “ggmap” v.3.0.0^29^ (https://github.com/dkahle/ggmap) and “ggraph” v.2.0.0^30^ (https://ggraph.data-imaginist.com).

**Figure 6:**
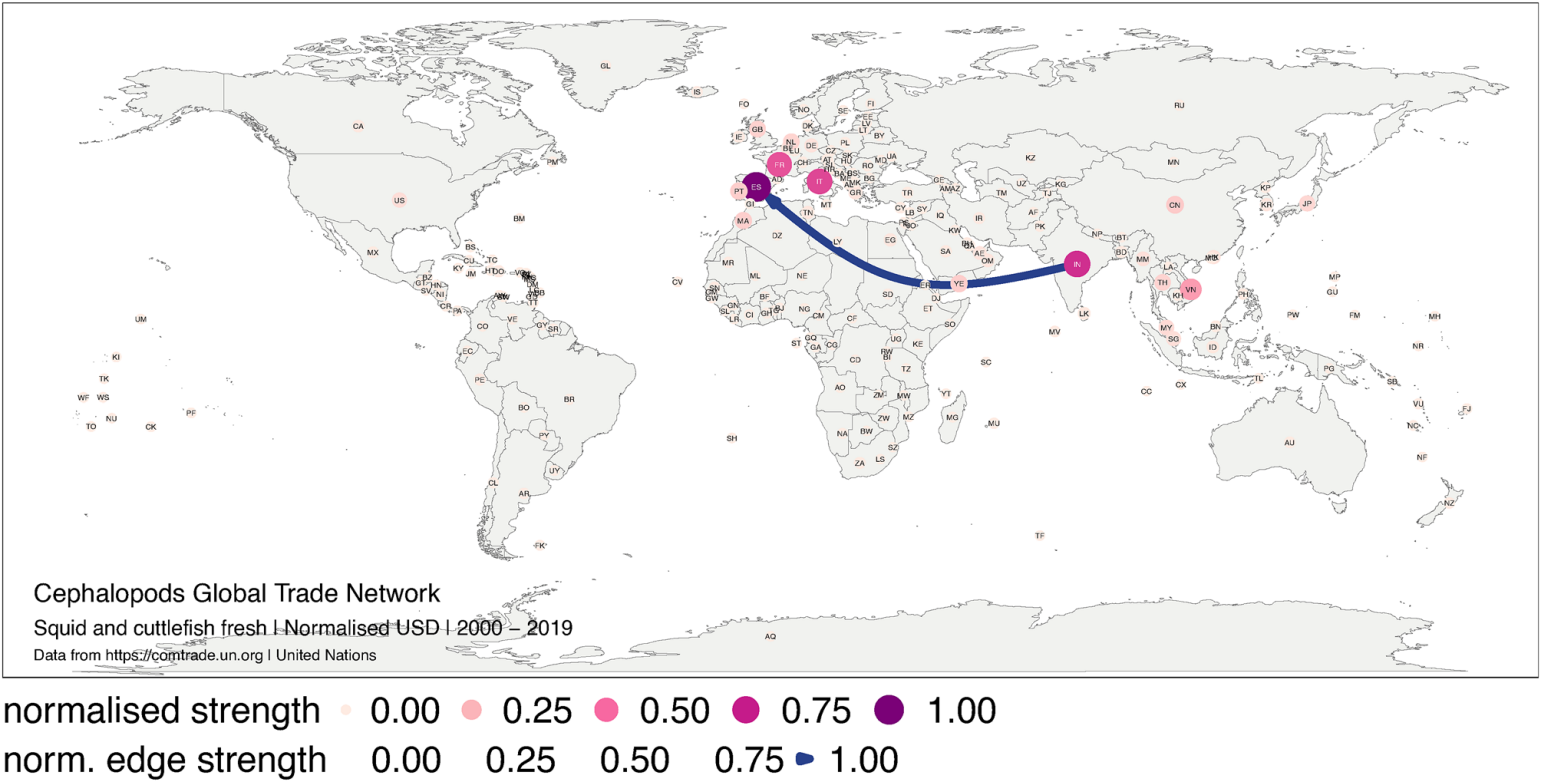
Global trade network for squid and cuttlefish live, fresh or chilled between 1 January 2000, and 31 December 2019 in monetary value (USD). The numbers correspond to the normalised strength for the monetary value. Each node represents a trader, and each edge represents the export–import relationship between two traders. The size and colour of the node represent the relative importance of the trader in the network in terms of its strength. The width and colour of the edge represent the relative importance of the relationship between two traders in terms of their edge strength. The figure was created with R^12^ (https://cran.r-project.org) packages: “ggplot2” v.3.2.1^13^ (https://ggplot2.tidyverse.org), “ggmap” v.3.0.0^29^ (https://github.com/dkahle/ggmap) and “ggraph” v.2.0.0^30^ (https://ggraph.data-imaginist.com).

The original Article has been corrected.

