# Supplementary material for: A network analysis of global cephalopod trade
Source: Sci Rep. 2022 Jan 10;12:322. doi: 10.1038/s41598-021-03777-9 (PMC8748611; doi:10.1038/s41598-021-03777-9)
Supplement: Supplementary file 1 — Supplementary Figures. [file 41598_2021_3777_MOESM1_ESM.pdf]

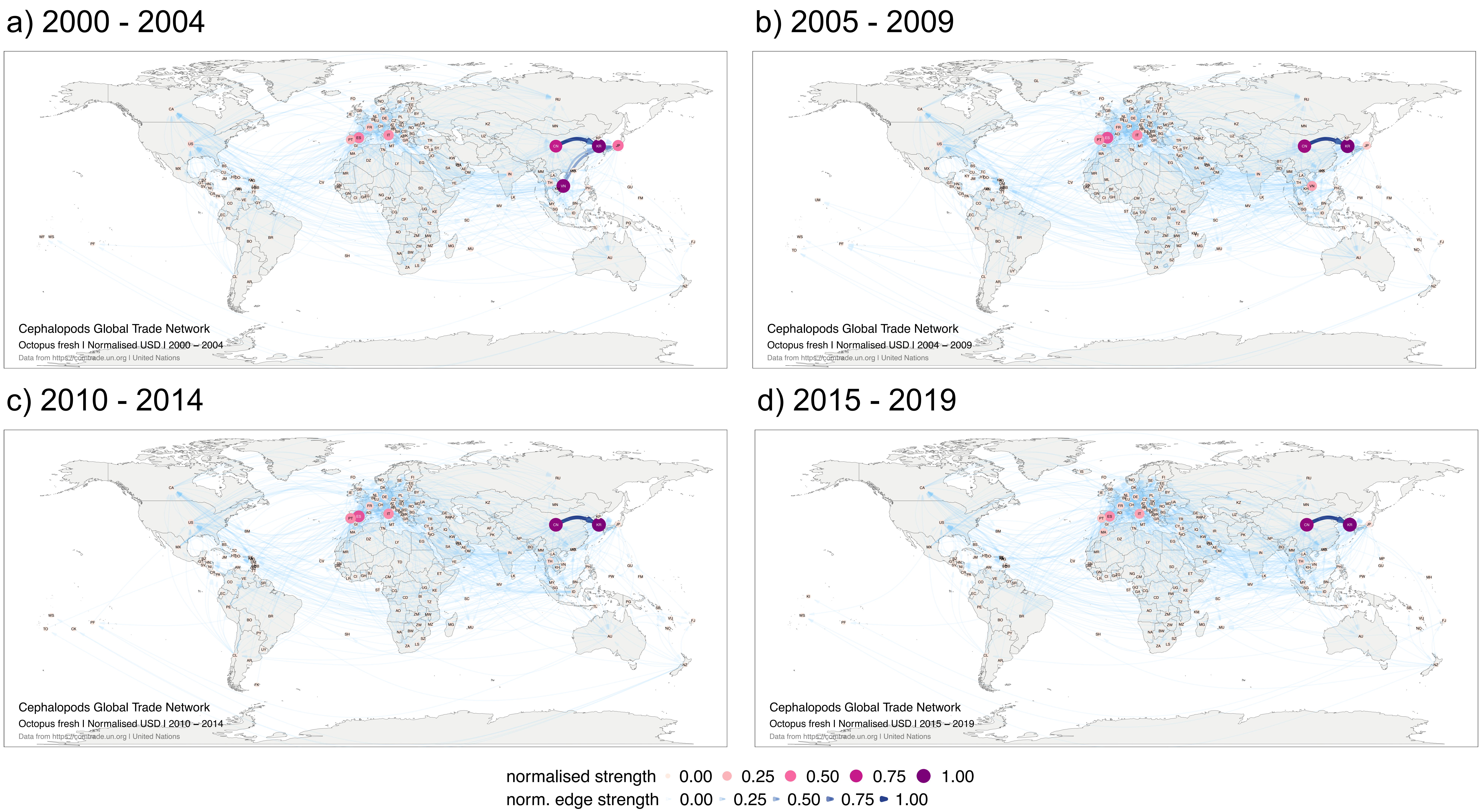

Supplementary Figure S1. Evolution of the global trade network for octopus live, fresh or chilled between 1 January 2000, and 31 December 2019 in monetary value (USD). Four five-year periods were considered. The numbers correspond to the normalised strength for the monetary value. Each node represents a trader, and each edge represents the export-import relationship between two traders. The size and colour of the node represent the relative importance of the trader in the network in terms of its strength. The width and colour of the edge represent the relative importance of the relationship between two traders in terms of their edge strength. The figure was created with R (https://cran.r-project.org) packages: “ggplot2” v.3.2.1 (https://ggplot2.tidyverse.org), “ggmap” v.3.0.0 (https://github.com/dkahle/ggmap) and “ggraph” v.2.0.0 (https://ggraph.data-imaginist.com).

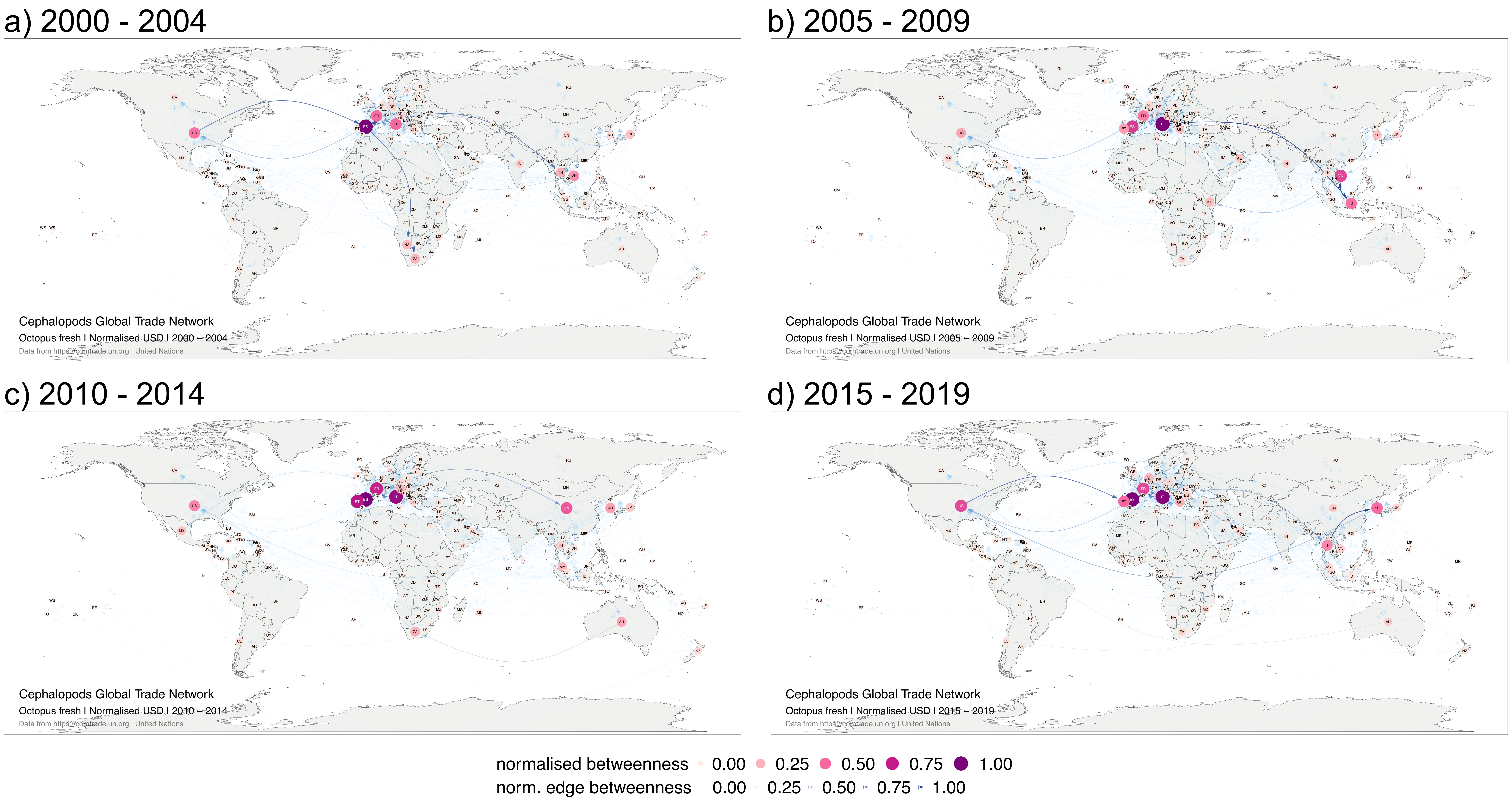

Supplementary Figure S2. Evolution of the global trade network for octopus live, fresh or chilled between 1 January 2000, and 31 December 2019 in monetary value (USD). Four five-year periods were considered. The numbers correspond to the normalised betweenness for the monetary value. Each node represents a trader, and each edge represents the relationship between two traders. The size and colour of the node represent the relative importance of the trader in the network in terms of its betweenness. The width and colour of the edge represent the relative importance of the relationship between two traders in terms of their edge betweenness. The figure was created with R (https://cran.r-project.org) packages: "ggplot2" v.3.2.1 (https://ggplot2.tidyverse.org), "ggmap" v.3.0.0 (https://github.com/dkahle/ggmap) and "ggraph" v.2.0.0 (https://ggraph.data-imaginist.com).

a) 2000 - 2009

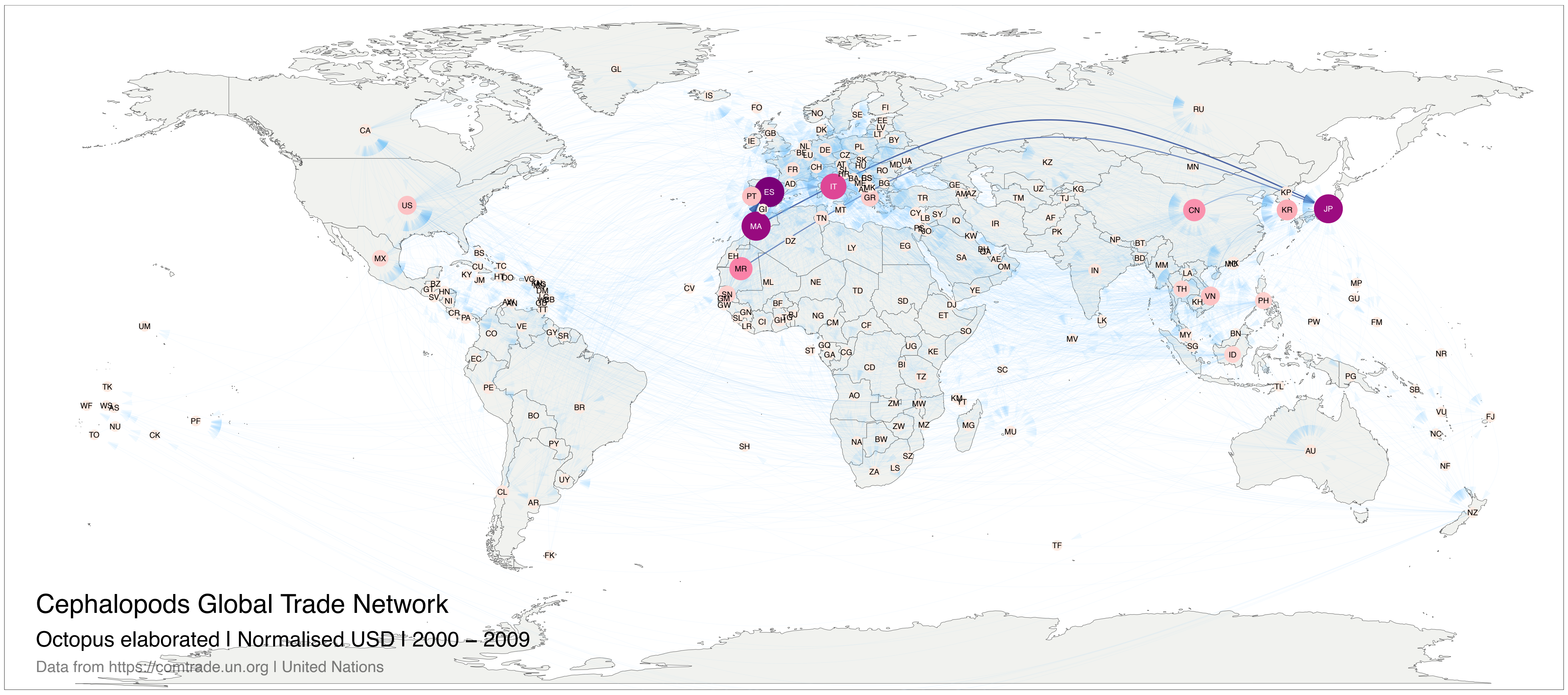

b) 2010 - 2019

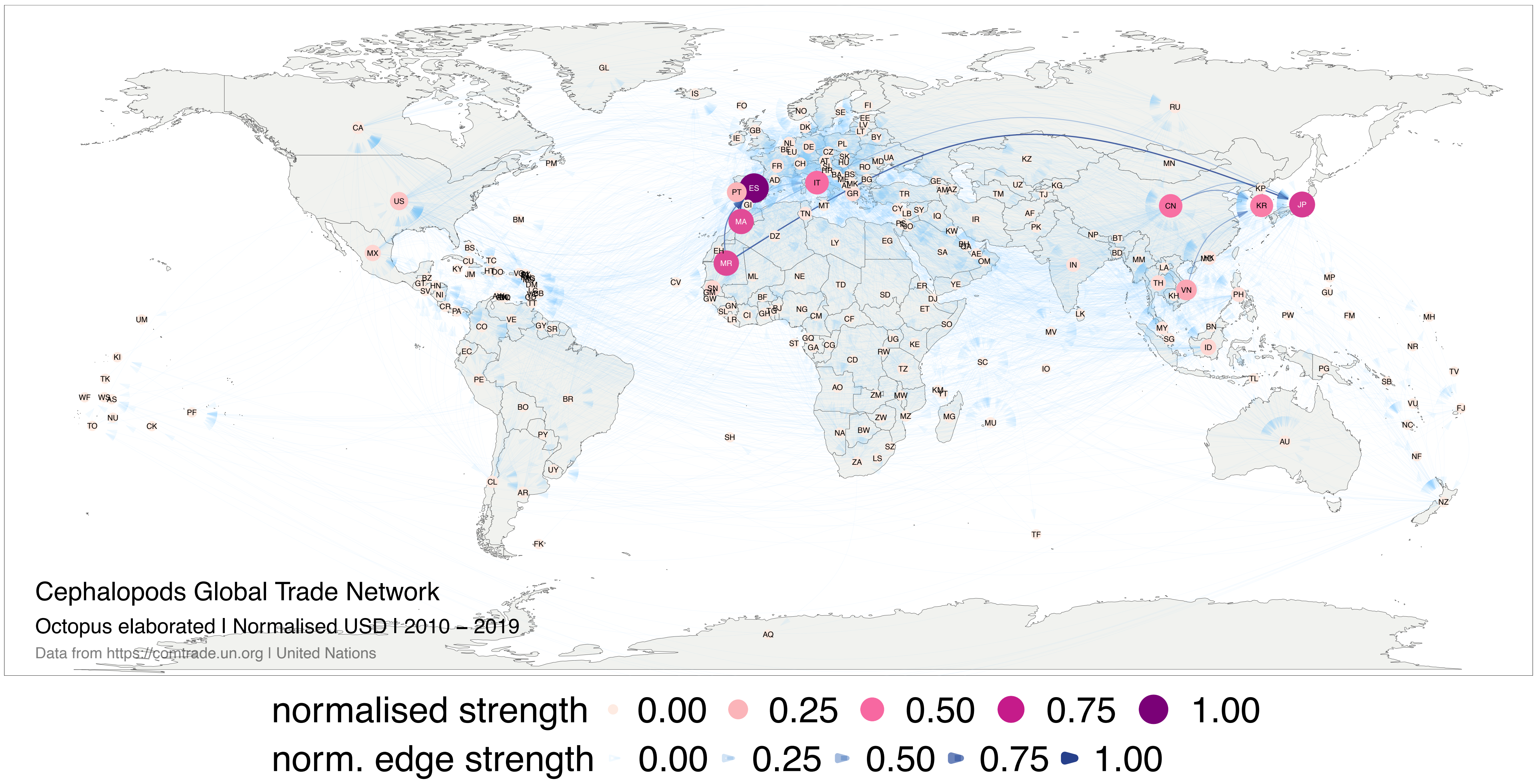

Supplementary Figure S3. Evolution of the global trade network for octopus elaborated between 1 January 2000, and 31 December 2019 in monetary value (USD). Two ten-year periods were considered. The numbers correspond to the normalised strength for the monetary value. Each node represents a trader, and each edge represents the export-import relationship between two traders. The size and colour of the node represent the relative importance of the trader in the network in terms of its strength. The width and colour of the edge represent the relative importance of the relationship between two traders in terms of their edge strength. The figure was created with R (https://cran.r-project.org) packages: “ggplot2” v.3.2.1 (https://ggplot2.tidyverse.org), “ggmap” v.3.0.0 (https://github.com/dkahle/ggmap) and “ggraph” v.2.0.0 (https://ggraph.data-imaginist.com).

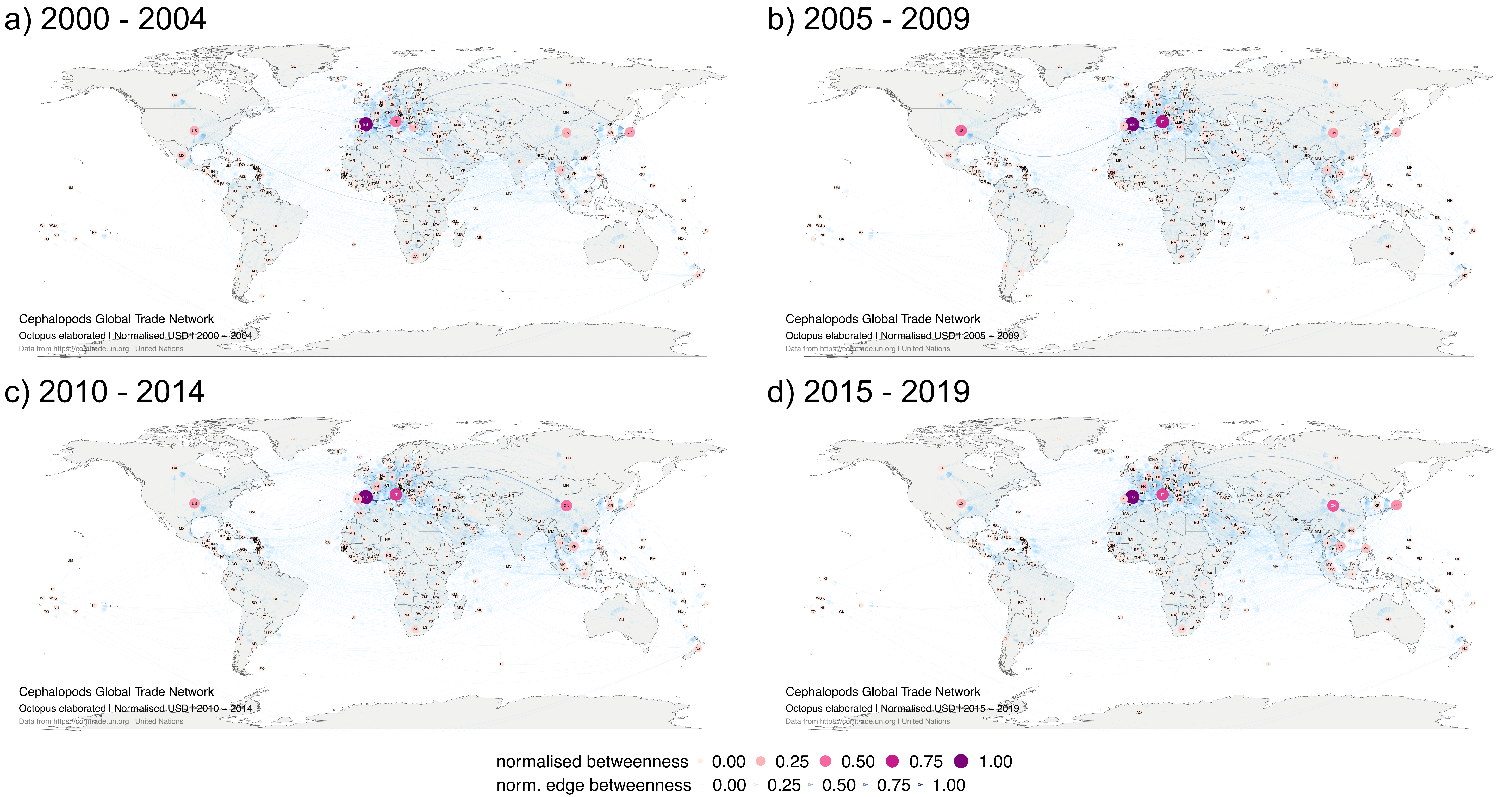

Supplementary Figure S4. Evolution of the global trade network for octopus elaborated between 1 January 2000, and 31 December 2019 in monetary value (USD). Four five-year periods were considered. The numbers correspond to the normalised betweenness for the monetary value. Each node represents a trader, and each edge represents the relationship between two traders. The size and colour of the node represent the relative importance of the trader in the network in terms of its betweenness. The width and colour of the edge represent the relative importance of the relationship between two traders in terms of their edge betweenness. The figure was created with R (https://cran.r-project.org) packages: “ggplot2” v.3.2.1 (https://ggplot2.tidyverse.org), “ggmap” v.3.0.0 (https://github.com/dkahle/ggmap) and “ggraph” v.2.0.0 (https://ggraph.data-imaginist.com).

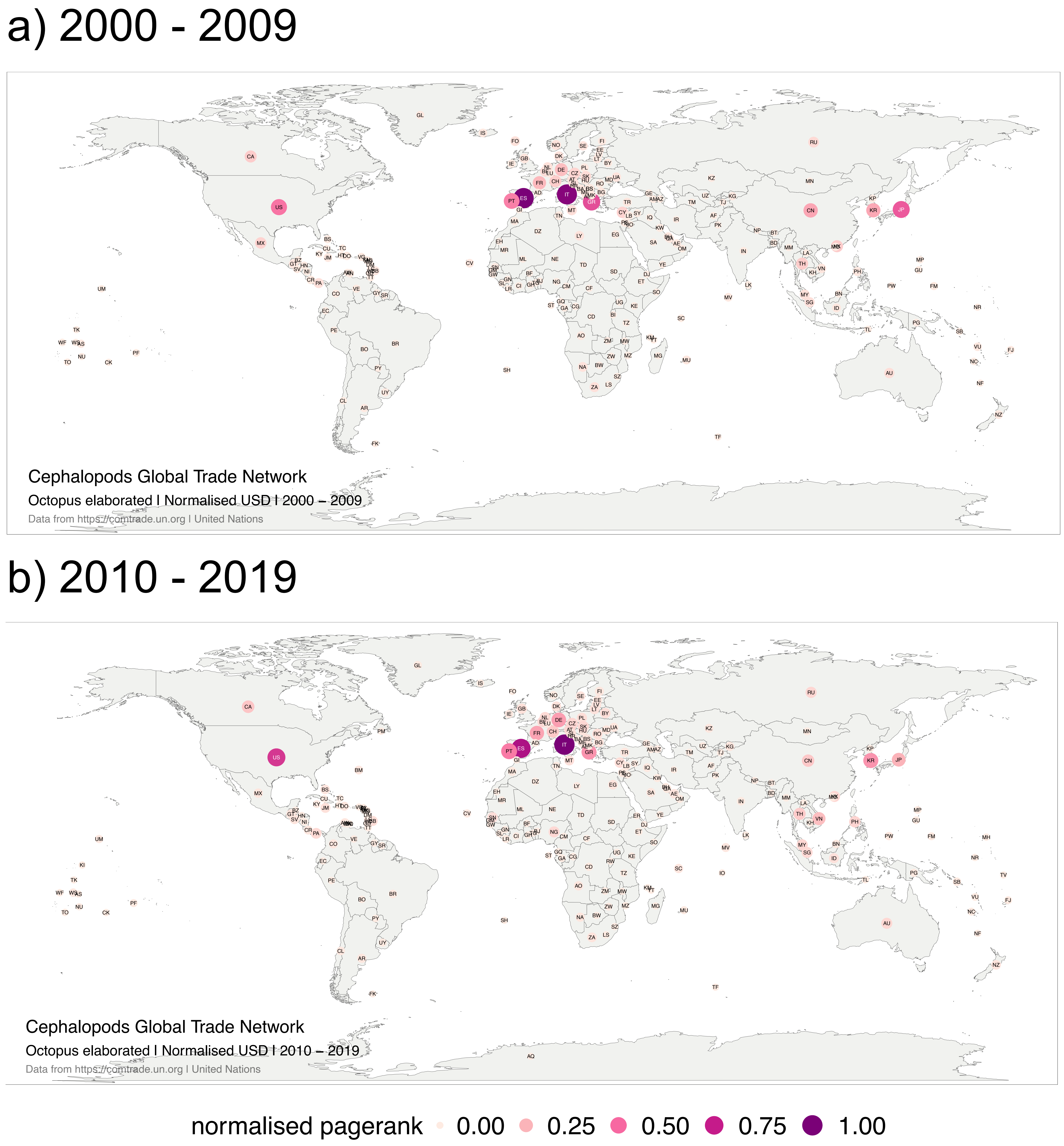

Supplementary Figure S5. Global trade network for octopus elaborated between 1 January 2000, and 31 December 2019 in monetary value (USD). Two ten-year periods were considered. The numbers correspond to the normalised PageRank for the monetary value. Each node represents a trader. The size and colour of the node represent the relative importance of the trader in the network in terms of its PageRank. The figure was created with R (https://cran.r-project.org) packages: “ggplot2” v.3.2.1 (https://ggplot2.tidyverse.org), “ggmap” v.3.0.0 (https://github.com/dkahle/ggmap) and “ggraph” v.2.0.0 (https://ggraph.data-imaginist.com).

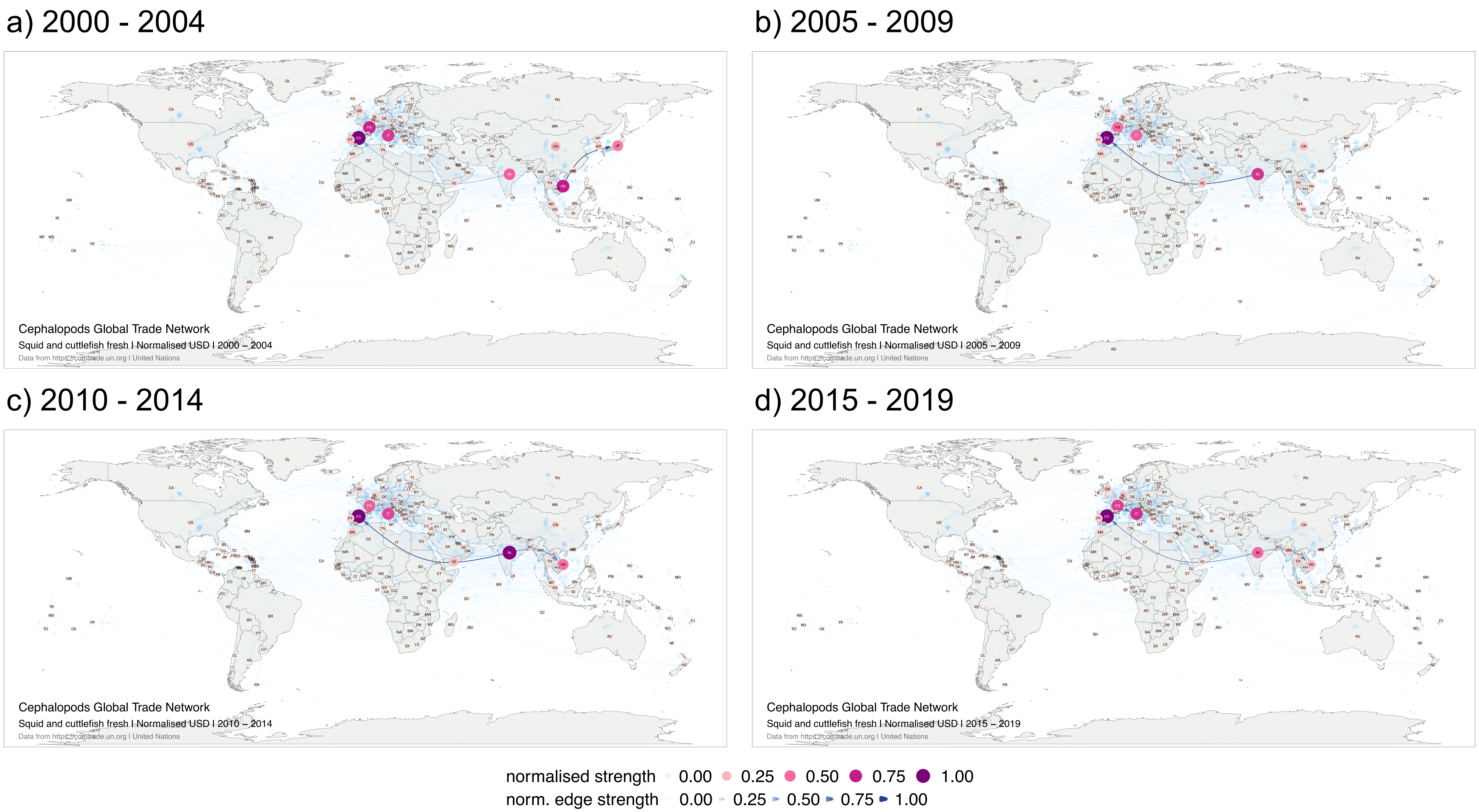

Supplementary Figure S6. Evolution of the global trade network for squid and cuttlefish live, fresh or chilled between 1 January 2000, and 31 December 2019 in monetary value (USD). Four five-year periods were considered. The numbers correspond to the normalised strength for the monetary value. Each node represents a trader, and each edge represents the export-import relationship between two traders. The size and colour of the node represent the relative importance of the trader in the network in terms of its strength. The width and colour of the edge represent the relative importance of the relationship between two traders in terms of their edge strength. The figure was created with R (https://cran.r-project.org) packages: “ggplot2” v.3.2.1 (https://ggplot2.tidyverse.org), “ggmap” v.3.0.0 (https://github.com/dkahle/ggmap) and “ggraph” v.2.0.0 (https://ggraph.data-imaginist.com).

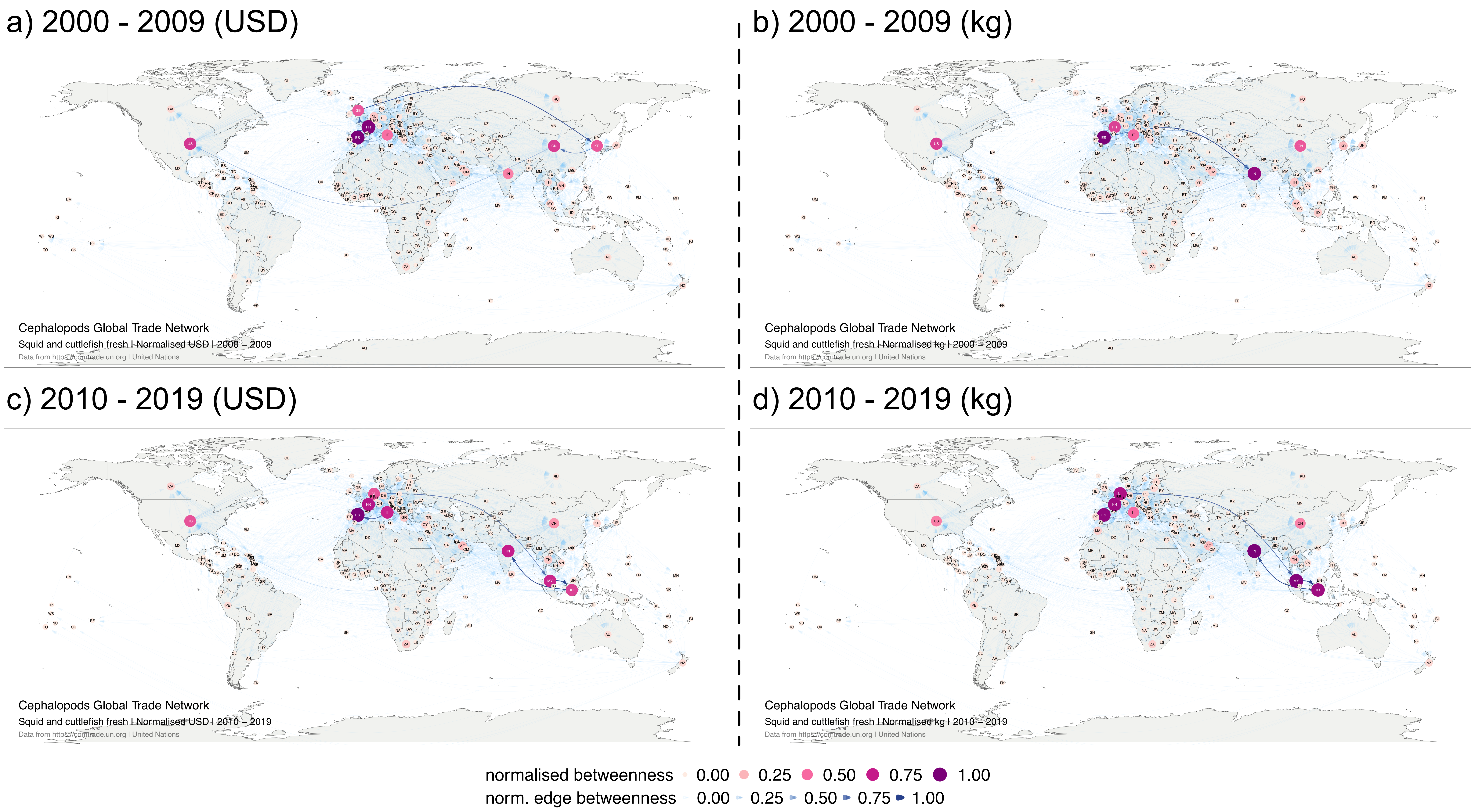

Supplementary Figure S7. Global trade network for squid and cuttlefish live, fresh or chilled between 1 January 2000, and 31 December 2019 in monetary value (USD) in the left-hand column, and volume (kg) in the right-hand column. The numbers correspond to the normalised betweenness for the monetary value (USD) and volume (kg) traded, respectively. Two ten-year periods have been considered, 2000-2009 in the top row and 2010-2019 in the bottom row. Each node represents a trader, and each edge represents the relationship between two traders. The size and colour of the node represent the relative importance of the trader in the network in terms of its betweenness. The width and colour of the edge represent the relative importance of the relationship between two traders in terms of their edge betweenness. The figure was created with R (https://cran.r-project.org) packages: “ggplot2” v.3.2.1 (https://ggplot2.tidyverse.org), “ggmap” v.3.0.0 (https://github.com/dkahle/ggmap) and “ggraph” v.2.0.0 (https://ggraph.data-imaginist.com).

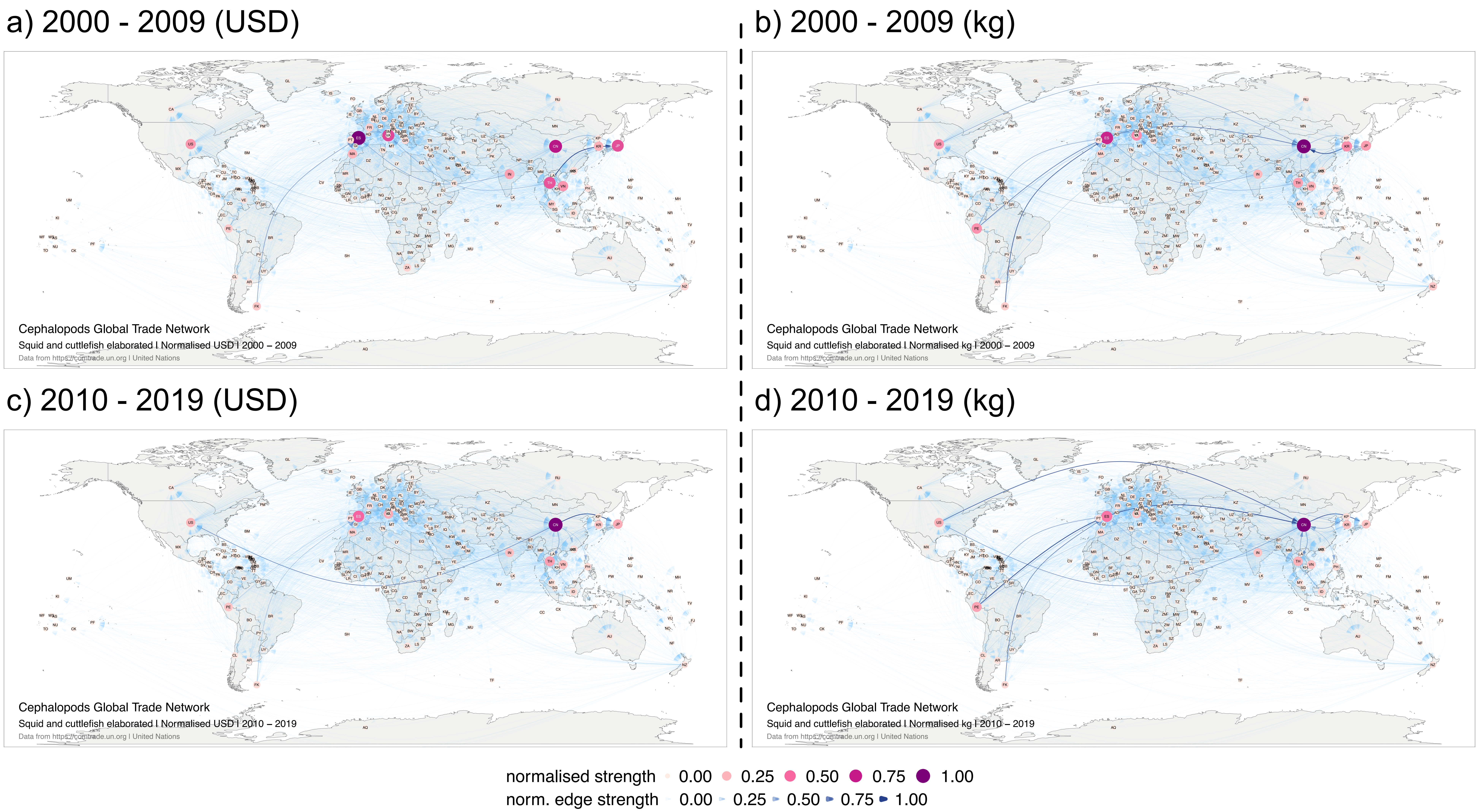

Supplementary Figure S8. Global trade network for squid and cuttlefish elaborated between 1 January 2000, and 31 December 2019 in monetary value (USD) in the left-hand column, and volume (kg) in the right-hand column. The numbers correspond to the normalised strength for the monetary value (USD) and volume (kg) traded, respectively. Two ten-year periods have been considered, 2000-2009 in the top row and 2010-2019 in the bottom row. Each node represents a trader, and each edge represents the relationship between two traders. The size and colour of the node represent the relative importance of the trader in the network in terms of its strength. The width and colour of the edge represent the relative importance of the relationship between two traders in terms of their edge strength. The figure was created with R (https://cran.r-project.org) packages: “ggplot2” v.3.2.1 (https://ggplot2.tidyverse.org), “ggmap” v.3.0.0 (https://github.com/dkahle/ggmap) and “ggraph” v.2.0.0 (https://ggraph.data-imaginist.com).

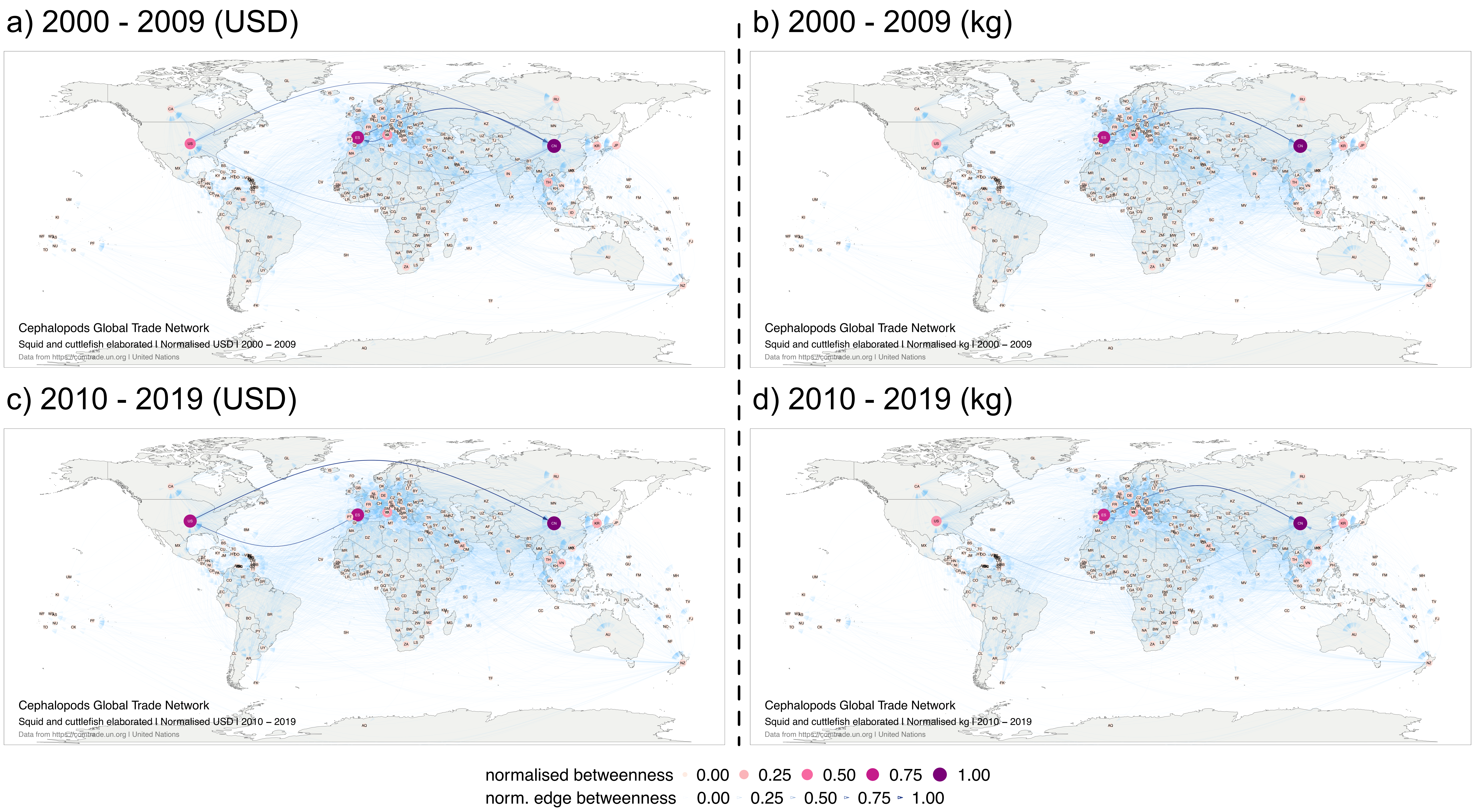

Supplementary Figure S9. Global trade network for squid and cuttlefish elaborated between 1 January 2000, and 31 December 2019 in monetary value (USD) in the left-hand column, and volume (kg) in the right-hand column. The numbers correspond to the normalised betweenness for the monetary value (USD) and volume (kg) traded, respectively. Two ten-year periods have been considered, 2000-2009 in the top row and 2010-2019 in the bottom row. Each node represents a trader, and each edge represents the relationship between two traders. The size and colour of the node represent the relative importance of the trader in the network in terms of its betweenness. The width and colour of the edge represent the relative importance of the relationship between two traders in terms of their edge betweenness. The figure was created with R (https://cran.r-project.org) packages: “ggplot2” v.3.2.1 (https://ggplot2.tidyverse.org), “ggmap” v.3.0.0 (https://github.com/dkahle/ggmap) and “ggraph” v.2.0.0 (https://ggraph.data-imaginist.com).

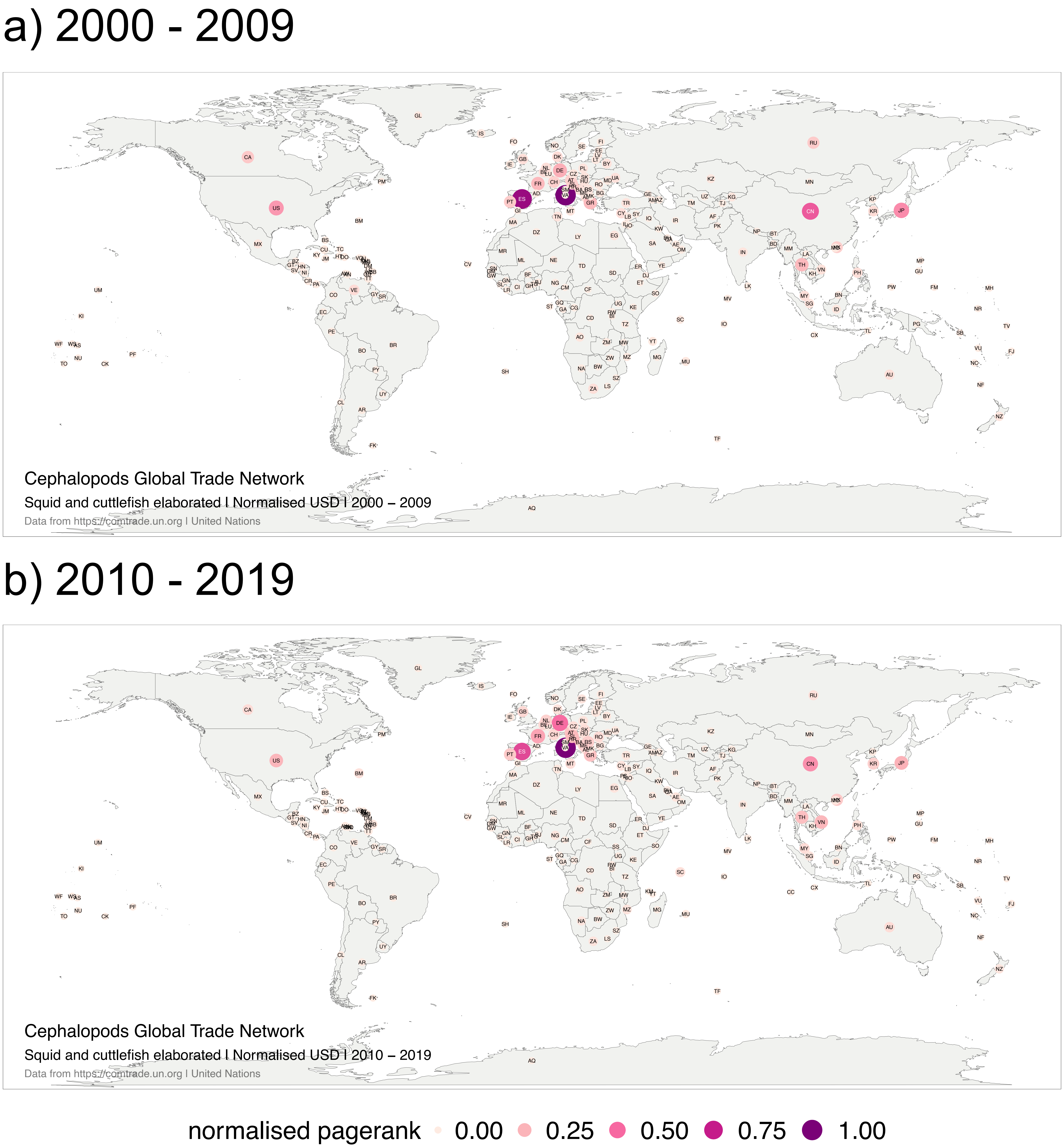

Supplementary Figure S10. Global trade network for squid and cuttlefish elaborated between 1 January 2000, and 31 December 2019 in monetary value (USD). Two ten-year periods were considered. The numbers correspond to the normalised PageRank for the monetary value. Each node represents a trader. The size and colour of the node represent the relative importance of the trader in the network in terms of its PageRank. The figure was created with R (https://cran.r-project.org) packages: "ggplot2" v.3.2.1 (https://ggplot2.tidyverse.org), "ggmap" v.3.0.0 (https://github.com/dkahle/ggmap) and "ggraph" v.2.0.0 (https://ggraph.data-imaginist.com).
