# Supplementary material for: A network analysis of global cephalopod trade
Source: Sci Rep. 2022 Jan 10;12:322. doi: 10.1038/s41598-021-03777-9 (PMC8748611; doi:10.1038/s41598-021-03777-9)
Supplement: Supplementary file 2 — Supplementary Tables. [file 41598_2021_3777_MOESM2_ESM.pdf]

Supplementary tables for the paper entitled:

A network analysis of the global cephalopod trade

Andres Ospina-Alvarez *; Silvia de Juan; Pablo Pita; Gillian Barbara Ainsworth; Fábio L. Matos; Cristina Pita; Sebastián Villasante

* Corresponding author: Andres Ospina-Alvarez aospina.co@me.com; Mediterranean Institute for Advanced Studies IMEDEA (UIB-CSIC), C/ Miquel Marques 21, CP 07190 Esporles, Balearic Islands, Spain.

Supplementary tables

Supplementary Table S1 – United Nations COMTRADE Harmonized System Codes and code descriptions that include octopuses, cuttlefish, and squids.

Species	Presentation	Code(s)	Description
**Octopuses**	live	030751	Molluscs; octopus (octopus spp.), live, fresh or chilled
	elaborated	030752<br>030759<br>160555	Molluscs; octopus (Octopus spp.), frozen, dried, salted, in brine, or smoked, cooked or not before or during the smoking process
**Cuttlefish and squids**	live	030741<br>030742	Molluscs; cuttle fish and squid, whether in shell or not, live, fresh or chilled
	elaborated	030743<br>030749<br>160554	Molluscs; cuttle fish and squid, whether in shell or not, includes flours, meals, and pellets of molluscs, fit for human consumption, frozen, dried, salted, in brine, or smoked, cooked or not before or during the smoking process

Supplementary Table S2 – Measures of centrality, definitions, and rationales in a context of a global trade network. See references for additional information.

Measure	Reference	Formal definition and equation	Plain language definition	Rationale in a global trade market
Strength	Barrat et al., 2004[1]; Squartini et al., 2013[2]	The Strength of a node *i* is defined as the sum of the weights of the in-coming links and the weights of the out-going links. $$strength_i = \sum_{j \neq i} w_{ij} + w_{ji}$$ where: w = weighted matrix w w_{ij} = weight of the directed link from node *i* to node *j* w_{ji} = weight of the directed link from node *j* to node *i*	Also named weighted degree. The node degree is the number of relations (edges) of the nodes. In weighted networks, node Strength is the sum of weights of links connected to the node.	Strength could indicate if a trader is involved in important (by weight) trades with other traders. Traders with high Strength can be acting as keystones since they are connected by imports and exports to many neighbouring traders.
In-strength	Squartini et al., 2013[2]	The In-strength of a node *i* is defined as the sum of the weights of the in-coming links. $$\mathrm{In} - strength_i = \sum_{j \neq i} w_{ij}$$ where: w = weighted matrix w w_{ij} = weight of the directed link from node *i* to node *j*	In directed networks, the In-strength is the sum of inward link weights.	Traders with a high in-strength could act as important importers or hubs for the distribution of raw materials. High in-strength could also be targeting traders acting as major consumers of products.
Out-strength	Squartini et al., 2013[2]	The Out-strength of a node *i* is defined as the sum of the weights of the out-going links. $$\mathrm{Out} - strength_i = \sum_{j \neq i} w_{ji}$$ where: w = weighted matrix w w_{ji} = weight of the directed link from node *j* to node *i*	In directed networks, the Out-strength is the sum of outward link weights.	Traders with high out-strength may be acting as raw producers with high export flows. This may indicate the geographical origin of the commodities and essential habitats for the species. Out-strength can also indicate whether a trader is a major exporter of processed products.

Closeness	Freeman, 1979[3]	Closeness centrality indicates how close a node is to all other nodes in the network. It is calculated as the average of the shortest path length from the node *i* to every other node in the network. $$C_i = \frac{1}{N-1}\sum_{j=1,\, j\neq i}^{N}\frac{1}{d_{ij}}$$ where: C_i = Closeness centrality of node *i* d_{ij} = distance between nodes *i* and *j* N = number of activity nodes in the network	Closeness centrality indicates how long it will take for information from a given node to reach other nodes in the network.	Traders with a higher closeness have a high probability of exporting to the nearest neighbouring traders. These traders could be important in trade at regional or continental geographic scales.
Betweenness	Freeman, 1979[3]	Betweenness centrality is calculated with the number of shortest paths (between any couple of nodes in the networks) that go through the target node *i*. The score is moderated through the total number of shortest paths between any pair of nodes in the network. The target node will have a high betweenness centrality if it appears in many shortest paths. $$B_i = \frac{1}{(N-1)\,(N-2)}\sum_{s,\, d=1,\, s\neq d\neq i}^{N}\frac{\sigma_{sd}\,(i)}{\sigma_{sd}}$$ σ_{sd} = number of shortest paths from source node *s* to destination node *d* $\sigma_{sd}\,(i)$ = number of those paths that include node *i*	Betweenness centrality is a measure of the influence of a node over the flow of information between every pair of nodes under the assumption that information primarily flows over the shortest paths.	Traders with high Betweenness centralities have been called "bottlenecks" or "bridges" and prevent network fragmentation. A trader that acts as a bridge between two well differentiated groups of traders usually has a high Betweenness.
Edge betweenness	Girvan and Newman, 2002[4]	Edge betweenness centrality is a measure of the centrality of an edge in a network based on the number of shortest paths through the given edge. $$\mathrm{EB_e} = \sum_{i}\sum_{j}\frac{\sigma_{ij}^{(e)}}{\sigma_{ij}}$$ EB_e = edge betweenness centrality of edge *e* σ_{ij} = number of shortest paths between nodes *i* and *j* $\sigma_{ij}^{(e)}$ = number of shortest paths between *i* and *j* that go through the edge *e*	Edge betweenness centrality identifies the edges of the network that are crucial for information flows.	An edge with a high edge betweenness centrality score represents a bridge-like connector between two countries or territories in the global market, and whose removal may affect the flow of goods between many pairs of partners through the shortest paths between them.

PageRank	Brin and Page 1998[5]	A variant of Eigenvector Centrality, primarily used for directed networks. PageRank considers (1) the number of in-coming links (i.e., nodes that link to a target node), (2) the quality of the linkers (i.e., the PageRank of nodes that link to the target node), and (3) the link propensity of the linkers (i.e., the number of nodes the linkers link to).<br><br>$$PR_i = 1 - \alpha + \alpha\left(\frac{PR_{t1}}{C_{t1}} + \ldots + \frac{PR_{tn}}{C_{tn}}\right)$$<br><br>where:<br>PR(i) = PageRank of a node *i*;<br>α = factor (estimated to be 0.85)<br>PR_{t1} to PR_{tn} = PageRank of nodes linking to *i*<br>C_{t1} to C_{tn} = number of outgoing links in those nodes	Algorithm developed by Larry Page and Sergey Brin, founders of Google. PageRank works by assigning importance to a webpage (node) if important pages (other nodes) point to it.	The most important traders are likely to import more products from other traders in the network. The approximate estimation of the importance of a trader is based on the number and quality (weight) of the links pointing to it. It indicates the trading relevance of the country providing the goods to another country.

Supplementary Table S3 – International merchandise trade information of octopus for the period 2000-2019 downloaded from United Nations COMTRADE Database[6]. Annual values and net weights (M USD and kg, respectively) for **exports** by presentation (1) live, fresh and chilled and (2) elaborated, (i.e., frozen, dried, salted or in brine) and country or territory, summarized by five-year periods are showed.

	**Octopus live, fresh or chilled**				**Octopus elaborated**			
**period**	**Exporter**	**M USD**	**Exporter**	**kg**	**Exporter**	**M USD**	**Exporter**	**kg**
2000-2004	VNM	165492	CHN	61977	MAR	1509626	MAR	355718
	CHN	125446	VNM	52940	MRT	547059	CHN	177555
	PRT	36880	IND	7552	ESP	526166	ESP	128029
	ESP	25351	ESP	6955	CHN	433574	MRT	117092
	FRA	18258	PRT	6452	PHL	238368	VNM	102808
2005-2009	CHN	224653	CHN	71875	MAR	1600080	MAR	236282
	ESP	72799	VNM	20486	MRT	911619	CHN	185217
	VNM	70659	ESP	13781	ESP	871620	MRT	146930
	PRT	68774	PRT	10415	CHN	622088	ESP	144553
	FRA	22882	IND	6260	VNM	436858	VNM	139051
2010-2014	CHN	304046	CHN	51895	MAR	1850608	MAR	256312
	ESP	103437	ESP	17826	MRT	1484975	CHN	214459
	PRT	96371	PRT	15619	CHN	1233304	MRT	213771
	FRA	26564	FRA	4451	ESP	814462	VNM	129484
	ITA	20936	THA	2885	VNM	604109	ESP	112463
2015-2019	CHN	505596	CHN	43905	MRT	3673373	MRT	420049
	ESP	131605	ESP	17145	MAR	3236167	MAR	355753
	MAR	72622	MAR	10461	CHN	2324771	CHN	339201
	PRT	62804	PRT	9379	ESP	1867819	VNM	246433
	THA	36102	THA	5821	VNM	1632688	ESP	209583

Supplementary Table S4 – International merchandise trade information of octopus for the period 2000-2019 downloaded from United Nations COMTRADE Database[6]. Annual values and net weights (M USD and kg, respectively) **imports** by presentation (1) live, fresh and chilled and (2) elaborated, (i.e., frozen, dried, salted or in brine) and country or territory, summarized by five-year periods are showed.

	**Octopus live, fresh or chilled**				**Octopus elaborated**			
**period**	**Importer**	**M USD**	**Importer**	**kg**	**Importer**	**M USD**	**Importer**	**kg**
2000-2004	KOR	175704	KOR	77741	JPN	1553028	JPN	371690
	JPN	74740	JPN	23836	ESP	784420	ITA	216423
	ITA	52451	ITA	17896	ITA	770489	KOR	188493
	ESP	51094	ESP	12033	KOR	325354	ESP	160514
	DEU	13466	USA	3783	USA	282802	USA	91573
2005-2009	KOR	260707	KOR	81405	JPN	1431980	ITA	257271
	ITA	84522	ITA	19921	ESP	1270921	JPN	244071
	ESP	81187	ESP	14582	ITA	1268764	KOR	229791
	PRT	27986	JPN	6452	KOR	607200	ESP	197773
	JPN	22255	PRT	5426	PRT	367077	USA	97058
2010-2014	KOR	323381	KOR	55243	JPN	2717785	JPN	357586
	ITA	103908	ITA	17376	ESP	2161674	KOR	321149
	ESP	88137	ESP	15171	ITA	1768354	ESP	308557
	PRT	30493	PRT	6197	KOR	1419080	ITA	285916
	FRA	11659	THA	2531	PRT	539837	USA	86226
2015-2019	KOR	547532	KOR	50122	ESP	3498286	ESP	375435
	ITA	145475	ITA	20193	JPN	2677873	KOR	361905
	ESP	80986	ESP	11755	KOR	2218955	JPN	313709
	PRT	39323	PRT	5779	ITA	2054556	ITA	282538
	JPN	21469	THA	3693	USA	976601	USA	134805

Supplementary Table S5 – International merchandise trade flow information of octopus for the period 2000-2019 downloaded from United Nations COMTRADE Database[6]. Annual values and net weights (M USD and kg, respectively) **flows** by presentation (1) live, fresh and chilled and (2) elaborated, (i.e., frozen, dried, salted or in brine), summarized by five-year periods are showed.

	**Octopus live, fresh or chilled**						**Octopus elaborated**					
**Period**	**From**	**To**	**M USD**	**From**	**To**	**kg**	**From**	**To**	**M USD**	**From**	**to**	**kg**
2000-2004	CHN	KOR	161745	CHN	KOR	74595	MAR	JPN	862546	MAR	JPN	202302
	VNM	JPN	91524	VNM	JPN	28796	MAR	ESP	733516	CHN	KOR	180411
	VNM	KOR	77894	VNM	KOR	24093	MRT	JPN	555171	MAR	ESP	127801
	PRT	ESP	45635	PRT	ESP	7922	CHN	KOR	325997	MRT	JPN	101454
	VNM	ITA	19915	VNM	ITA	6177	MAR	ITA	292246	PHL	USA	80626
2005-2009	CHN	KOR	234785	CHN	KOR	68135	MAR	ESP	1065527	CHN	KOR	175640
	PRT	ESP	72919	PRT	ESP	11300	MRT	JPN	1033992	MRT	JPN	127167
	ESP	ITA	38496	ESP	ITA	6810	CHN	KOR	602106	MAR	ESP	123739
	ESP	PRT	30552	ESP	PRT	5889	MAR	ITA	504015	VNM	KOR	111947
	FRA	ITA	17602	FRA	ITA	3618	MAR	JPN	498350	MAR	ITA	72336
2010-2014	CHN	KOR	251726	CHN	KOR	40822	MAR	ESP	1004817	CHN	KOR	145122
	PRT	ESP	60217	PRT	ESP	9989	MRT	JPN	765279	MAR	ESP	132459
	ESP	ITA	52263	ESP	ITA	8436	CHN	KOR	669114	MRT	JPN	114401
	ESP	PRT	21575	ESP	PRT	4176	MAR	JPN	590790	MAR	JPN	93747
	FRA	ITA	17416	THA	KOR	2722	CHN	JPN	502503	VNM	KOR	85193
2015-2019	CHN	KOR	504888	CHN	KOR	43698	MRT	ESP	1206486	MRT	ESP	116638
	ESP	ITA	78577	ESP	ITA	10266	MAR	ESP	901392	VNM	KOR	90599
	PRT	ESP	53243	PRT	ESP	8275	MRT	JPN	750224	MRT	JPN	77704
	THA	KOR	35526	THA	KOR	5578	VNM	KOR	659975	CHN	KOR	77015
	ESP	PRT	26236	ESP	PRT	3900	CHN	KOR	477015	MAR	ESP	71617

Supplementary Table S6 – International merchandise trade information of squid and cuttlefish for the period 2000-2019 downloaded from United Nations COMTRADE Database[6]. Annual values and net weights (M USD and kg, respectively) **exports** by presentation (1) live, fresh and chilled and (2) elaborated, (i.e., frozen, dried, salted or in brine) and country or territory, summarized by five-year periods are showed.

	**Squid and cuttlefish live, fresh or chilled**				**Squid and cuttlefish elaborated**			
**Period**	**Exporter**	**M USD**	**Exporter**	**kg**	**Exporter**	**M USD**	**Exporter**	**kg**
2000-2004	VNM	263274	VNM	111305	THA	1218713	CHN	353754
	FRA	208438	IND	81698	CHN	853376	VNM	352478
	IND	186148	FRA	50396	VNM	840921	THA	348019
	ESP	106946	ESP	38524	ESP	644729	PER	321395
	GBR	37699	MYS	24398	IND	633827	KOR	314145
2005-2009	IND	381356	IND	119806	THA	1544010	PER	840925
	FRA	217535	ESP	38752	CHN	1521777	CHN	580167
	ESP	137293	FRA	35714	IND	1159411	KOR	487542
	YEM	91532	YEM	32386	VNM	1144073	IND	403873
	MAR	70877	MMR	27662	MAR	851123	VNM	348854
2010-2014	IND	660746	IND	176121	CHN	4422557	CHN	992110
	FRA	324540	YEM	58010	IND	1571034	PER	712759
	ESP	206466	ESP	49039	THA	1354423	USA	521380
	YEM	171032	FRA	47530	MAR	1267262	IND	460789
	MAR	108062	MYS	24510	PER	1067929	ESP	265760
2015-2019	IND	550503	IND	140866	CHN	13031464	CHN	2659384
	FRA	401225	ESP	69874	PER	2973330	PER	1312285
	ESP	335056	MMR	60954	IND	2909037	IND	733147
	MAR	112520	FRA	55420	ESP	2409776	ESP	591112
	GBR	111487	ARE	29528	VNM	1896376	IDN	510551

Supplementary Table S7 – International merchandise trade information of squid and cuttlefish for the period 2000-2019 downloaded from United Nations COMTRADE Database[6]. Annual values and net weights (M USD and kg, respectively) **imports** by presentation (1) live, fresh and chilled and (2) elaborated, (i.e., frozen, dried, salted or in brine) and country or territory, summarized by five-year periods are showed.

	**Squid and cuttlefish live, fresh or chilled**				**Squid and cuttlefish elaborated**			
**Period**	**Importer**	**M USD**	**Importer**	**kg**	**Importer**	**M USD**	**Importer**	**kg**
2000-2004	ESP	246587	ESP	76874	ESP	1665014	CHN	869754
	ITA	220339	JPN	65308	JPN	1644186	ESP	744793
	JPN	149236	ITA	62506	ITA	1369386	ITA	536940
	CHN	78701	CHN	51732	CHN	857777	JPN	473081
	PRT	54815	PRT	23237	USA	476655	USA	227518
2005-2009	ESP	537952	ESP	114909	ESP	2699191	CHN	1325208
	ITA	343014	ITA	71734	ITA	2102858	ESP	930600
	CHN	104544	THA	53331	JPN	1727261	ITA	621562
	FRA	76725	CHN	48298	CHN	1370368	JPN	453326
	PRT	61852	SGP	26958	USA	793441	USA	278719
2010-2014	ESP	570329	VNM	115843	ESP	3520585	CHN	1592914
	ITA	477699	ESP	101359	ITA	2653687	ESP	956054
	VNM	369784	ITA	86497	CHN	2467566	ITA	585550
	FRA	97691	THA	25227	JPN	2394740	THA	508099
	PRT	70699	FRA	25093	USA	1554431	JPN	437191
2015-2019	ITA	473583	ITA	79904	ESP	4833683	CHN	1348976
	ESP	441469	ESP	70031	JPN	3637286	ESP	1182812
	VNM	156951	THA	57778	ITA	3484044	THA	703432
	FRA	130257	VNM	49741	CHN	3074877	JPN	662119
	THA	86803	FRA	26932	THA	2839169	ITA	660693

Supplementary Table S8 – International merchandise trade flow information of squid and cuttlefish for the period 2000-2019 downloaded from United Nations COMTRADE Database[6]. Annual values and net weights (M USD and kg, respectively) **flows** by presentation (1) live, fresh and chilled and (2) elaborated, (i.e., frozen, dried, salted or in brine), summarized by five-year periods are showed.

	**Squid and cuttlefish live, fresh or chilled**						**Squid and cuttlefish elaborated**					
**Period**	**from**	**to**	**M USD**	**from**	**to**	**kg**	**from**	**to**	**M USD**	**from**	**to**	**kg**
2000-2004	VNM	JPN	139700	VNM	JPN	58286	THA	JPN	877954	FLK	ESP	266591
	FRA	ITA	103108	IND	ESP	33003	FLK	ESP	523186	KOR	CHN	195707
	FRA	ESP	91729	FRA	ITA	24009	ESP	ITA	516108	ESP	ITA	192089
	IND	ESP	88851	FRA	ESP	23147	CHN	JPN	498743	THA	ITA	157968
	ESP	ITA	37014	MYS	SGP	19675	MAR	ESP	447100	CHN	JPN	157299
2005-2009	IND	ESP	257496	IND	ESP	68183	MAR	ESP	896992	KOR	CHN	279419
	FRA	ITA	128389	MMR	THA	36090	THA	JPN	621124	USA	CHN	265627
	FRA	ESP	121727	MYS	SGP	25374	IND	ESP	602311	PER	CHN	249362
	MAR	ESP	94135	FRA	ITA	22034	FLK	ESP	532354	FLK	ESP	195523
	ESP	ITA	80041	IND	ITA	19816	CHN	USA	515489	MAR	ESP	168430
2010-2014	IND	ESP	251142	IND	VNM	68329	CHN	JPN	954841	PER	CHN	333451
	IND	VNM	212893	IND	ESP	54152	CHN	USA	904261	USA	CHN	251325
	FRA	ITA	163393	YEM	VNM	27798	MAR	ESP	854224	PRK	CHN	199927
	FRA	ESP	144221	IND	ITA	27540	CHN	THA	827695	CHN	JPN	188013
	ESP	ITA	124271	ESP	ITA	23678	CHN	HKG	764117	CHN	THA	183718
2015-2019	ESP	ITA	156190	MMR	THA	52612	CHN	JPN	1859101	CHN	JPN	322779
	FRA	ITA	154255	IND	VNM	35392	CHN	THA	1212316	IDN	CHN	260943
	FRA	ESP	153324	ESP	ITA	29896	CHN	USA	1115636	FLK	ESP	235551
	IND	ESP	124374	IND	ESP	26842	CHN	PHL	831339	CHN	THA	217230
	IND	VNM	120331	FRA	ITA	21609	FLK	ESP	794218	PER	CHN	209072

References

1. Barrat, A., Barthélemy, M., Pastor-Satorras, R. & Vespignani, A. The architecture of complex weighted networks. *Proceedings of the National Academy of Sciences of the United States of America* **101**, 3747–3752 (2004).
2. Squartini, T., Picciolo, F., Ruzzenenti, F. & Garlaschelli, D. Reciprocity of weighted networks. *Sci Rep* **3**, 2729 (2013).
3. Freeman, L. C. Centrality in social networks conceptual clarification. *Social Networks* **1**, 215–239 (1979).
4. Girvan, M. & Newman, M. Community structure in social and biological networks. *Proceedings of the National Academy of Sciences of the United States of America* **99**, 7821–7826 (2002).
5. Brin, S. & Page, L. The anatomy of a large-scale hypertextual web search engine. *Computer Networks* **30**, 107–117 (1998).
6. Reister, M. & Muryawan, M. Quantity and weight data in UN Comtrade. https://unstats.un.org/wiki/display/comtrade/Quantity+and+Weight+Data+in+UN+Comtrade (2009).
